# Click chemistry-facilitated comprehensive identification of proteins adducted by antimicrobial 5-nitroimidazoles for discovery of alternative drug targets against giardiasis

**DOI:** 10.1371/journal.pntd.0008224

**Published:** 2020-04-17

**Authors:** Tineke Lauwaet, Yukiko Miyamoto, Sozaburo Ihara, Christine Le, Jarosław Kalisiak, Keith A. Korthals, Majid Ghassemian, Diane K. Smith, K. Barry Sharpless, Valery V. Fokin, Lars Eckmann

**Affiliations:** 1 Department of Pathology, University of California, San Diego, La Jolla, California, United States of America; 2 Department of Medicine, University of California, San Diego, La Jolla, California, United States of America; 3 Division of Gastroenterology, The Institute for Adult Diseases, Asahi Life Foundation, Tokyo, Japan; 4 Department of Chemistry and The Skaggs Institute for Chemical Biology, The Scripps Research Institute, La Jolla, California, United States of America; 5 Department of Chemistry and Biochemistry, University of California, San Diego, La Jolla, California, United States of America; 6 Department of Chemistry and Biochemistry, San Diego State University, San Diego, California, United States of America; 7 Department of Chemistry, Dornsife College of Letters, Arts and Sciences, University of Southern California, Los Angeles, California, United States of America; University of Texas at El Paso, UNITED STATES

## Abstract

Giardiasis and other protozoan infections are major worldwide causes of morbidity and mortality, yet development of new antimicrobial agents with improved efficacy and ability to override increasingly common drug resistance remains a major challenge. Antimicrobial drug development typically proceeds by broad functional screens of large chemical libraries or hypothesis-driven exploration of single microbial targets, but both strategies have challenges that have limited the introduction of new antimicrobials. Here, we describe an alternative drug development strategy that identifies a sufficient but manageable number of promising targets, while reducing the risk of pursuing targets of unproven value. The strategy is based on defining and exploiting the incompletely understood adduction targets of 5-nitroimidazoles, which are proven antimicrobials against a wide range of anaerobic protozoan and bacterial pathogens. Comprehensive adductome analysis by modified click chemistry and multi-dimensional proteomics were applied to the model pathogen *Giardia lamblia* to identify dozens of adducted protein targets common to both 5’-nitroimidazole-sensitive and -resistant cells. The list was highly enriched for known targets in *G*. *lamblia*, including arginine deiminase, α-tubulin, carbamate kinase, and heat shock protein 90, demonstrating the utility of the approach. Importantly, over twenty potential novel drug targets were identified. Inhibitors of two representative new targets, NADP-specific glutamate dehydrogenase and peroxiredoxin, were found to have significant antigiardial activity. Furthermore, all the identified targets remained available in resistant cells, since giardicidal activity of the respective inhibitors was not impacted by resistance to 5’-nitroimidazoles. These results demonstrate that the combined use of click chemistry and proteomics has the potential to reveal alternative drug targets for overcoming antimicrobial drug resistance in protozoan parasites.

## Author summary

*Giardia lamblia* and other protozoan parasites annually infect hundreds of millions of individuals worldwide and are major causes of clinically important diseases. Vaccines against giardiasis are not available, making treatment with antimicrobial drugs critical for the management of the infection. Resistance to all major drugs against *G*. *lamblia* has been reported and threatens their future utility, yet development of new and improved drugs remains a major scientific and economic challenge. Here we report a strategy for identifying new drug targets by exploring the targets of a successful class of existing antimicrobial drugs with multiple targets. Comprehensive identification of these targets revealed possibilities for alternative drugs that can overcome existing forms of resistance. This strategy has the potential to accelerate identification of promising novel drug targets as key rate-limiting step in the development of alternative antimicrobial drugs.

## Introduction

5-Nitroimidazoles (NI) comprise an important class of antimicrobial drugs extensively employed in the treatment of protozoal infections [1]. Metronidazole (Mz), first developed in the 1950s and approved by the FDA in 1963, is the most widely used 5-NI drug worldwide and is included in the WHO list of essential medicines. Mz is highly active in vitro and in vivo against clinically important pathogenic protozoa which annually infect hundreds of millions of individuals worldwide. Most prominent among these protozoa are *Giardia lamblia*, a major cause of protracted diarrheal disease and growth retardation in children in the developing world [2], and *Entamoeba histolytica*, which can cause dysentery and liver abscesses [3]. In addition, Mz is employed against *Trichomonas vaginalis*, a sexually transmitted infection that causes vaginitis, adverse pregnancy outcomes, increased susceptibility to HIV, and cervical and prostate cancers [4,5], and several important anaerobic bacterial pathogens, particularly *Helicobacter pylori* and *Clostridium difficile* [1].

Mz and other 5-NIs are prodrugs that act in a two-step fashion. First, they are reduced and thereby activated by low redox potential reactions mediated by ferredoxin, nitroreductases, or thioredoxin reductases in the target microbes [6,7]. Second, several of the reactive intermediates, such as the nitroso and hydroxylamine forms of the prodrugs, can then react with target molecules to form adducts that may lead to target inactivation [8]. Reactive intermediates may also be inactivated themselves by further reduction or reoxidation if sufficient oxygen is available before they can react with microbial target molecules or potentially host molecules. The formation and relative abundance of different reactive and non-reactive nitro drug intermediates is poorly characterized, partly due to their unstable nature, and may vary from microbe to microbe [9]. Although the general outline of 5-NI activation and the resulting microbial selectivity are well understood, less is known about specific 5-NI drug targets and their respective roles in mediating antimicrobial 5-NI activity. Several target proteins of Mz have been identified by two-dimensional gel electrophoresis in *E*. *histolytica* (thioredoxin, thioredoxin reductase, superoxide dismutase, and purine nucleoside phosphorylase) [10] and *G*. *lamblia* (including thioredoxin reductase, giardins, branched-chain amino acid transferase, and pyruvate phosphate dikinase) [11]. In bacteria and protozoa, 5-NI drugs can also cause DNA damage [12,13], but the importance of this mechanism for antigiardial activity remains to be determined [13]. A different nitro-heterocyclic compound (NBDHEX) with activity against *G*. *lamblia* has been shown to form covalent adducts with select target proteins, including thioredoxin reductase and elongation factor 1B-γ, and structural proteins such as α-tubulin [14].

Despite the general efficacy of 5-NI drugs, treatment failures and drug resistance occur in a significant number of giardiasis cases [6,15–18] and have also been demonstrated for other protozoa [6,15,19], indicating an ongoing need for development of alternative antimicrobials against these pathogens. Resistance is thought to be related to downregulation or loss of drug-activating pathways, or increased activity of detoxifying pathways [6,16]. For example, diminished Mz activation can be related in *G*. *lamblia* to reduced expression of ferredoxin [20], which acts as an electron shuttle for 5-NI reduction, or pyruvate ferredoxin oxidoreductase [21], which maintains ferredoxin in a reduced state [22]. Similarly, decreased activity of two other 5-NI reducing enzymes, nitroreductase and thioredoxin reductase, can contribute to resistance [7,23,24]. In contrast, little evidence exists that downstream targets of activated 5-NI drugs can be involved in drug resistance, suggesting that a better understanding of these targets may be exploitable for guiding new drug development towards targets that have proven utility and are not compromised by nitro drug resistance. To test this hypothesis, we performed a comprehensive analysis of protein targets adducted by 5-NI drugs, using a newly developed click chemistry-based proteomics approach and *G*. *lamblia* as a model system. We demonstrate that the results can serve as a basis for alternative drug discovery strategies.

## Methods

### Ethics statement

Animal care and use for this study was approved by the University of California, San Diego Institutional Animal Care and Use Committee under protocol number S00205 and United States Public Health Service assurance numbers A3033-1 and D16-00020. Animal use adhered to the guidelines in the most recent edition of the Guide for the Care and Use of Laboratory Animals of the National Research Council of the United States National Academies and the Guidelines on Euthanasia by the American Veterinary Medical Association.

### General chemical methods

All chemical reagents and solvents were purchased from commercial suppliers and used without further purification. ^1^H and ^13^C NMR spectra were obtained on a Varian Inova 400 MHz spectrometer. Chemical shifts (δ) were expressed in ppm relative to residual: CHCl_3_ (δH 7.26 ppm), CDCl_3_ (δC 77.0 ppm). Abbreviations are: s, singlet; t, triplet; m, multiplet. Reactions were monitored by LC-MS analysis (Hewlett-Packard Series 1100, ESI MS) eluting with 0.1% TFA in H_2_O and 0.05% TFA in CH_3_CN and/or TLC chromatography using Merck TLC Silica Gel 60 F254 plates and visualized by staining with cerium molybdate (Hanessian’s Stain) or by absorbance of UV light at 254 nm. Crude reaction mixtures were purified by column chromatography using Merck Silica Gel 60 as stationary phase.

### Synthesis of Mz-alkyne

1-(But-3-ynyl)-2-methyl-5-nitro-1H-imidazole (Mz-alkyne) was generated by reacting 1-(methoxymethyl)-2-methyl-4-nitro-1H-imidazole with but-3-ynyl trifluoromethanesulfonate, followed by removal of the methoxymethyl acetal protective group under acidic conditions. Briefly, 1-(methoxymethyl)-2-methyl-4-nitro-1*H*-imidazole was obtained as described for a similar compound [25]; ^1^H NMR (400 MHz, CDCl_3_) δ = 7.78 (s, 1H), 5.23 (s, 2H), 3.35 (s, 3H), 2.48 (s, 3H); ^13^C NMR (100 MHz, CDCl_3_) δ = 145.4, 119.7, 77.8, 56.6, 13.0; ESI MS (MeOH) for [M+Na]^+^ = 194.4 Da. A solution of this compound (12 g, 70.2 mmol) in CH_3_NO_2_ (50 ml) was added to but-3-ynyl trifluoromethanesulfonate [26] (15.6 g, 77.2 mmol, 1.1 equiv.) in CH_3_NO_2_ (50 ml), and the mixture was stirred overnight at 70°C. After evaporation, the residue was dissolved in 1 M HCl (110 mL), stirred at 70°C for 3 h, and cooled to room temperature. The reaction mixture was neutralized with 2 M NaOH and extracted using EtOAc. The organic layer was dried over MgSO_4_, filtered and evaporated, and the residue was purified by column chromatography (silica-gel, Et_2_O:MeOH, 95:5) to yield Mz-alkyne (5.2 g, 56%); ^1^H NMR (400 MHz, CDCl_3_) δ = 7.92 (s, 1H), 4.44 (t, *J* = 6.4 Hz, 2H), 2.73–2.69 (m, 2H), 2.54 (s, 3H), 2.01–1.99 (m, 1H); ^13^C NMR (100 MHz, CDCl_3_) δ = 150.9, 138.2, 133.1, 79.0, 71.7, 44.4, 19.9, 14.6; ESI MS (MeOH) for [M+H]^+^ = 180.4 Da.

### Synthesis of azido-biotin

For the synthesis of azido-biotin, a 20 mL scintillation vial was charged with biotin 4-nitrophenyl ester (0.561 g, 1.53 mmoles), 11-azido-3,6,9-trioxaundecan-1-amine (0.40 ml, 0.44 g, 2.02 mmol), and dry dichloromethane (10 ml). The solution was stirred overnight at room temperature, divided into four aliquots, and purified via Biotage using a Snap 10g cartridge running 2% methanol/dichloromethane (1% acetic acid) to 10% methanol/dichloromethane (1% acetic acid). After purification and isolation, a yield of 90% (0.612 g, 1.38 mmoles) was obtained. The spectroscopic characterization was consistent with prior reports [27]; mass spectrum m/z (% rel intensity, ESI+) 445 [M+1]+ (100).

### Parasites

Trophozoites of *G*. *lamblia* strain WB clone C6 (ATCC 50803) and a congenic Mz-resistant line [28], and strain GS/M (ATCC 50581) were cultured in modified TYI-S-33 medium with bovine bile and calf serum.

### Drug assays

The following inhibitors were purchased: Canavanine and metronidazole (Sigma Aldrich); vincristine, epigallocatechin gallate, and SNX-2112 (Selleck Chemicals); disulfiram (Tocris); and conoidin A (Cayman Chemical). For assays of giardicidal activity, serial 1:3 dilutions of these compounds were made in 96-well plates, *G*. *lamblia* WB or GS/M trophozoites were added, and cultures were grown for 1–2 days at 37°C under anaerobic conditions [29]. Parasite cell growth and viability were determined by measuring ATP levels with the BacTiter-Glo microbial cell viability assay reagent (Promega) in a microplate reader. The 50% effective concentration (EC50) was derived from the concentration-response curves using BioAssay software (Cambridge soft). Acute human cytotoxicity was assayed with the human epithelial cell line, HeLa (ATCC CCL-2) [29,30]. Compounds were serially diluted (1:3) and added to HeLa cell cultures in 96-well plates. Cells were grown for two days, and viable cell numbers were determined using AlamarBlue reagent (Invitrogen). As done for the EC50 calculations, the 50% cytotoxic concentration (CC50) was derived from the normalized concentration-response curves using BioAssay software (Cambridge soft).

### Click chemistry

Live *G*. *lamblia* WB trophozoites were treated with various concentrations of Mz-alkyne or DMSO as a solvent control, washed twice with ice-cold PBS, and lysed in 0.1 M ammonium bicarbonate containing 0.2% SDS and proteinase inhibitor cocktail (Roche) for 10 min at room temperature. Lysates were centrifuged for 5 min at 9,000 rpm, and supernatants (150 μg protein/300 μl) were incubated for 2 h at room temperature with the following click reaction mixture: 1.5 μM azido-biotin, 5 mM ascorbic acid, 2 mM tris[(1-tert-butyl-1H-1,2,3-triazolyl)methyl]amine, and 0.5 mM CuSO_4_ [31]. Samples were then processed for affinity purification or boiled in 5 x Laemmli buffer containing 100 mM DTT for immunoblotting. For cell free adduction assays, lysates of untreated *G*. *lamblia* WB trophozoites were prepared and incubated for 2 h with 500 μM Mz-alkyne and 1 mM dithionite prior to performing the click reaction.

### ELISA and immunoblotting

For ELISA of total protein adduction, cell lysates were reacted with azido-biotin by the click reaction, and coated to 96-well plates (Immulon 4HBX) over a range of protein amounts (2.5 μg-1.25 ng/well) in a bicarbonate buffer (pH 9.5) at 4°C overnight. Plates were washed three times with PBS + 0.05% Tween 20, incubated with streptavidin-HRP diluted in PBS/Tween 20. After washing the plate, signals were revealed by adding 100 μl of SureBlue reagent (VWR), and reactions were stopped with 100 μl of 1.2 M H_2_SO_4_. Optical density was measured at 450 nm in a Synergy microplate reader with the Gen5 Microplate Data Collection and Analysis Software (BioTek Instruments, Germany).

For gel electrophoresis, proteins were separated by 4–20% SDS-PAGE and transferred onto PVDF membranes, which were blocked with 3% milk in PBS/0.1% Tween-20 for 30 min, and reacted with azido-biotin by the click reaction and stained for 1 h with HRP-labeled anti-biotin or HRP-labeled streptavidin (Cell Signaling). After washing with PBS/Tween, HRP was visualized by chemiluminescence (ECL-plus, GE Healthcare). Immunoblot detection of ornithine carbamoyltransferase (OCT), arginine deiminase (ADI), and enolase in *G*. *lamblia* extracts was done as previously described [32,33].

### In vitro immunofluorescence analysis

Live trophozoites were allowed to attach to coverslips for 10 min at 37°C, washed in warm PBS, incubated with Mz-alkyne for 15 min, fixed in ice-cold methanol for 10 min, dried, and permeabilized with 0.5% Triton X-100 for 10 min. Fixed cells were incubated with click reaction mixture (described above) for 2 h at room temperature, washed with PBS, and blocked for 30 min in 5% goat serum, 1% glycerol, 0.1% BSA, 0.1% fish gelatin and 0.04% sodium azide. Cells were subsequently incubated for 1 h with Alexa 488-labeled anti-biotin (Life Technologies), washed, post-fixed with 4% paraformaldehyde, rinsed with water, and mounted with Prolong Gold with DAPI (Molecular Probes). Staining was examined at 1,000 x magnification using a Nikon Eclipse E800 microscope equipped with an X-Cite 120 fluorescence lamp. Confocal images were taken with a Leica TCS SP5 system attached to a Leica DMI 6000 inverted microscope.

### Murine *G*. *lamblia* infections

For in situ staining experiments, suckling (5 day-old) female C57 mice (The Jackson Laboratory) were infected by oral gavage with 1 × 10^7^ *G*. *lamblia* WB clone C6 trophozoites. On day 4, mice were orally given 100 mg/kg Mz-alkyne suspended in 0.1% methylcellulose in PBS. After 2 h, the small intestine was collected and opened longitudinally, embedded in Optimal Cutting Temperature compound, and snap-frozen in dry ice/isopropanol. Frozen sections (5 μm) were prepared, fixed in ice-cold methanol, permeabilized with 0.5% Triton X-100, stained by in situ click reaction with azido-biotin followed by Alexa 488-labeled anti-biotin antibody. Trophozoites were detected by co-staining with a rabbit antibody against the parasite-specific *Giardia* axoneme-associated 180 kDa protein (GASP-180) [34], followed by washing and incubation with Alexa 568-labeled anti-rabbit antibody (Life Technologies), and nuclear counterstaining with DAPI. Slides were examined by fluorescence microscopy (Nikon Eclipse E800 equipped with an X-Cite 120 fluorescence lamp).

For in vivo efficacy tests, male and female C57BL/6 mice (The Jackson Laboratory) were infected by oral gavage with 1 x 10^6^ *G*. *lamblia* GS/M trophozoites. Starting on day 1, mice were given canavanine (Sigma-Aldrich) at a dose of 2 g/kg for a total of three doses, or conoidin A (Cayman Chemicals) at a dose of 100 mg/kg for a total of four doses, in 0.1% hypromellose in PBS by oral gavage over a 3-day period. Controls received only hypromellose-PBS. On day 5, animals were euthanized by CO_2_ inhalation followed by cervical dislocation, the small intestine was removed, opened lengthwise in 5 ml PBS, and chilled and shaken. Live detached trophozoites were enumerated in a counting chamber. No significant differences were observed between males and females, and the data were pooled.

### Affinity purification and LC-MS/MS analysis of adducted proteins

After completion of the click reaction, proteins were precipitated with cold acetone (-20°C, 30 min), centrifuged (9,000 rpm, 5 min), and resuspended in an ammonium bicarbonate/SDS buffer at 300 μg per 600 μl. Biotin-labeled proteins were incubated with 75 μl streptavidin beads (Life Technologies) for 14 h at 4°C, and beads were pelleted (8,000 rpm, 5 min), washed, and stored at -80°C until analysis.

Protein-bead complexes were diluted in TNE (50 mM Tris pH 8.0, 100 mM NaCl, 1 mM EDTA) buffer. RapiGest SF reagent (Waters Corp.) was added to the mix to a final concentration of 0.1% and samples were boiled for 5 min. TCEP [Tris (2-carboxyethyl) phosphine] was added to 1 mM (final concentration) and the samples were incubated at 37°C for 30 min. Subsequently, samples were carboxymethylated with 0.5 mg/ml of iodoacetamide for 30 min at 37°C, followed by neutralization with 2 mM TCEP (final concentration) and digestion with trypsin (trypsin:protein ratio of 1:50) overnight at 37°C. RapiGest was degraded and removed by treating the samples with 250 mM HCl at 37°C for 1 h followed by centrifugation at 15,800 *g* for 30 min at 4°C. The soluble fraction was then added to a new tube, and peptides were extracted and desalted using Aspire RP30 desalting columns (Thermo Scientific).

Trypsin-digested peptides were analyzed by high pressure liquid chromatography coupled with tandem mass spectroscopy (LC-MS/MS) using nanospray ionization as described [35] with the following modifications: The nanospray ionization experiments were performed using a QSTAR-Elite hybrid mass spectrometer (ABSCIEX) interfaced with nano-scale reversed-phase HPLC (Tempo) using a 10 cm-100 micron ID glass capillary packed with 5-μm C18 Zorbax beads (Agilent Technologies, Santa Clara, CA). Peptides were eluted from the C18 column into the mass spectrometer using a linear gradient (5–60%) of acetonitrile (ACN) at a flow rate of 400 μl/min for 1 h. The buffers used to create the ACN gradient were: Buffer A (98% H_2_O, 2% ACN, 0.2% formic acid, and 0.005% TFA) and Buffer B (100% ACN, 0.2% formic acid, and 0.005% TFA).

MS/MS data were acquired in a data-dependent manner in which the MS1 data were acquired at m/z of 400 to 1,800 Da and the MS/MS data were acquired from m/z of 50 to 2,000 Da. The MS was set to disregard ions from certain highly abundant non-giardial proteins (e.g. human keratin, streptavidin). Peptide identifications were made using the Paragon Algorithm, which combines database searches with sequence tag approaches [36], as implemented in the Protein Pilot 2.0 software package (Sciex). The software conducts integrated false discovery rate analysis for accurate assessment of the reliability of results. A comprehensive library of *G*. *lamblia* protein sequences was obtained from www.giardiadb/org. Relative abundance of adducted proteins was determined as normalized spectral abundance factors (NSAF) from spectral peptide counts, target protein size, and total number of detections [37].

### ADI production and structural analysis of nitro drug adduction

The gene encoding ADI (GenBank ID XP_001705755.1) was amplified from *G*. *lamblia* WB DNA by PCR using the primers 5’-GAA GGA GAT ATA CAT ATG ACT GAC TTC TCC AAG GAT AAA-3’ and 5’-GTG ATG GTG GTG ATG ATG CTT GAT ATC GAC GCA GAT GTC-3’. Mutations C283A and C442A were generated in the ADI gene by overlapping-extension PCR. The products were sequenced to confirm the presence of the desired mutations, and cloned into the bacterial expression vector pETite C-His (Lucigen). Bacterial expression vectors were individually transformed into BL21-CodonPlus(DE3)-RIL cells (Agilent), and protein expression was induced by addition of 1 mM IPTG. Recombinant proteins were purified from bacterial lysates using HisExpress columns (Claremont Biosolutions) according to the manufacturer’s instructions. Activity of ADI was assayed with a modified colorimetric citrulline-detection assay [38]. Total cell lysates (2 μg) or affinity purified ADI-HA from trophozoites treated or not with Mz-alkyne, Mz or iodoacetamide, or recombinant His-tagged ADI (0.5 μg) treated or not with 500 μM Mz-alkyne and 1 mM dithionite, or citrulline standards were incubated with 1 mM arginine and 75 mM Hepes (pH 7.0) in a 100 μl volume for 15 min at 37°C. Samples were boiled for 5 min in 1 ml of 16% H_2_SO_4_, 13% H_3_PO_4_, 16% FeCl_3_, 1.65% diacetyl monooxime and 1.3% thiosemicarbazide, and absorbance was determined at 530 nm. ADI protein structure was modeled by comparison to the resolved 3D structure of ADI in *P*. *aeruginosa* [39] using 3D-JIGSAW [40], and visualized with Polyview 3D [41].

### Statistical analysis

Unpaired t-test was used to analyze pEC50s of the drug assays, and Mann–Whitney U test was used for analysis of in vivo efficacy assays, with p<0.05 considered statistically significant. Data are presented as mean ± SD or SE for the indicated number of experiments. For the analysis of adducted target proteins, NSAF are shown as mean ± SD, and significance was determined by t-test with the null hypothesis of no adduction.

## Results

### Synthesis and testing of an alkyne-labeled 5-NI compound

**Fig 1:**
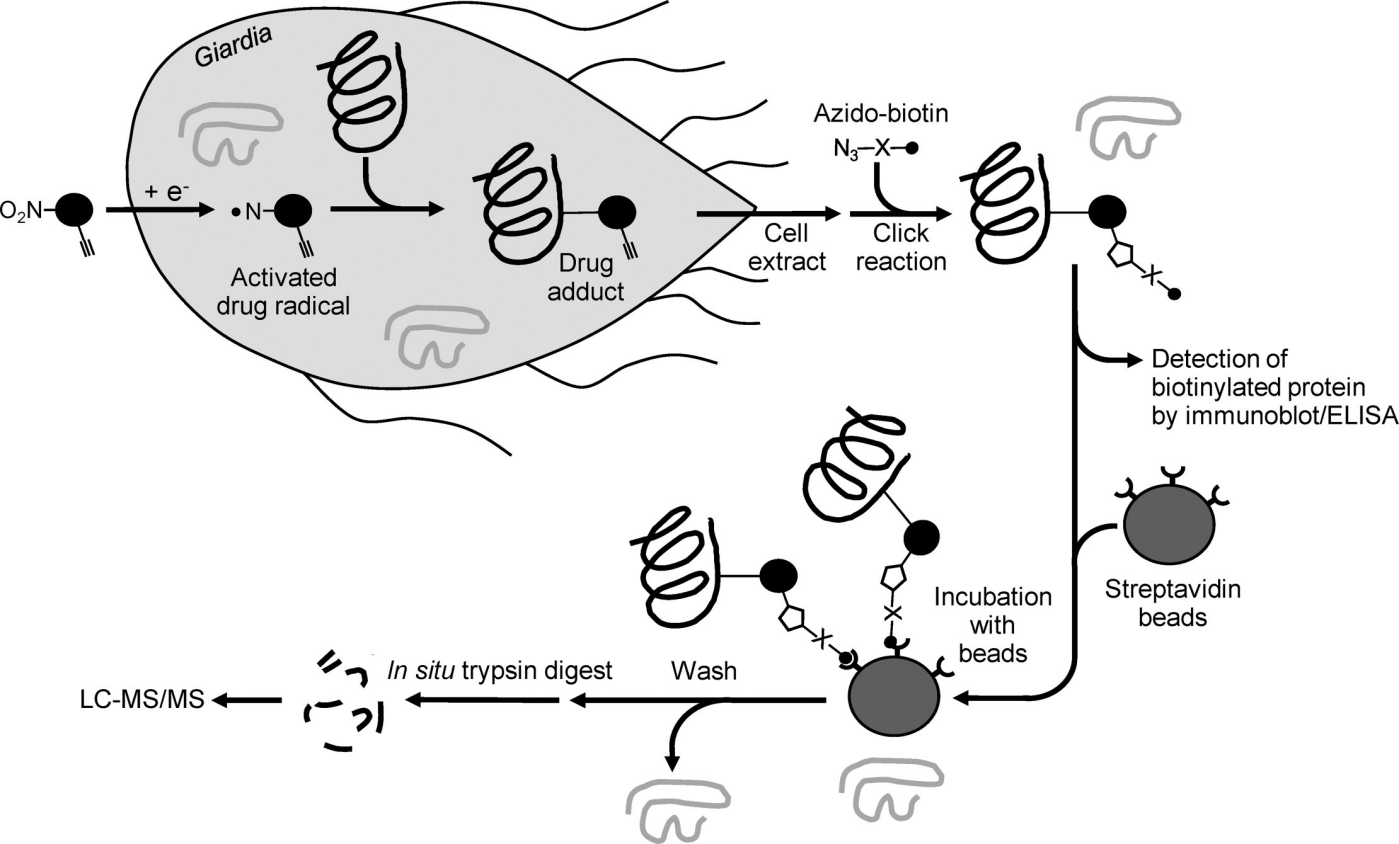
Strategy for identifying adduction targets of nitro drugs by click chemistry. An alkyne labeled 5-NI compound enters trophozoites as an inactive prodrug and is reduced to reactive intermediates, which form covalently linked adducts with multiple target molecules. Cell extracts are prepared and reacted with azido-biotin by the click reaction. The adducted and now biotin-labeled molecules can be detected by immunoblotting or ELISA, or can be isolated by streptavidin-affinity purification. Purified proteins are digested in situ with trypsin, and the resulting peptides are analyzed by LC-MS/MS.

5-NI drugs form covalently linked adducts with multiple target molecules. We reasoned that this stable linkage, when combined with a chemical strategy to selectively isolate adducted molecules (Fig 1), could be exploited to identify individual targets of this drug class and apply those insights to the development of new drug classes with more selective mechanisms of action. Key to this approach was addition of a small chemical tag to the 5-nitro drug that would not interfere with bioactivity in live cells, but could be used for subsequent target purification from cell extracts. To this end, we employed an adaptation of the copper(I) catalyzed azide-alkyne conjugation process (“click reaction”), in which a terminal alkyne is joined to an organic azide to form a stable 1,2,3-triazole ring in the presence of a copper(I) catalyst [31]. Because both functional groups are small and relatively inert in biological systems, and the click reaction proceeds under mild aqueous conditions and is quantitative, we hypothesized that this approach could be used for detection and identification of proteins adducted by 5-nitro drugs.

**Fig 2:**
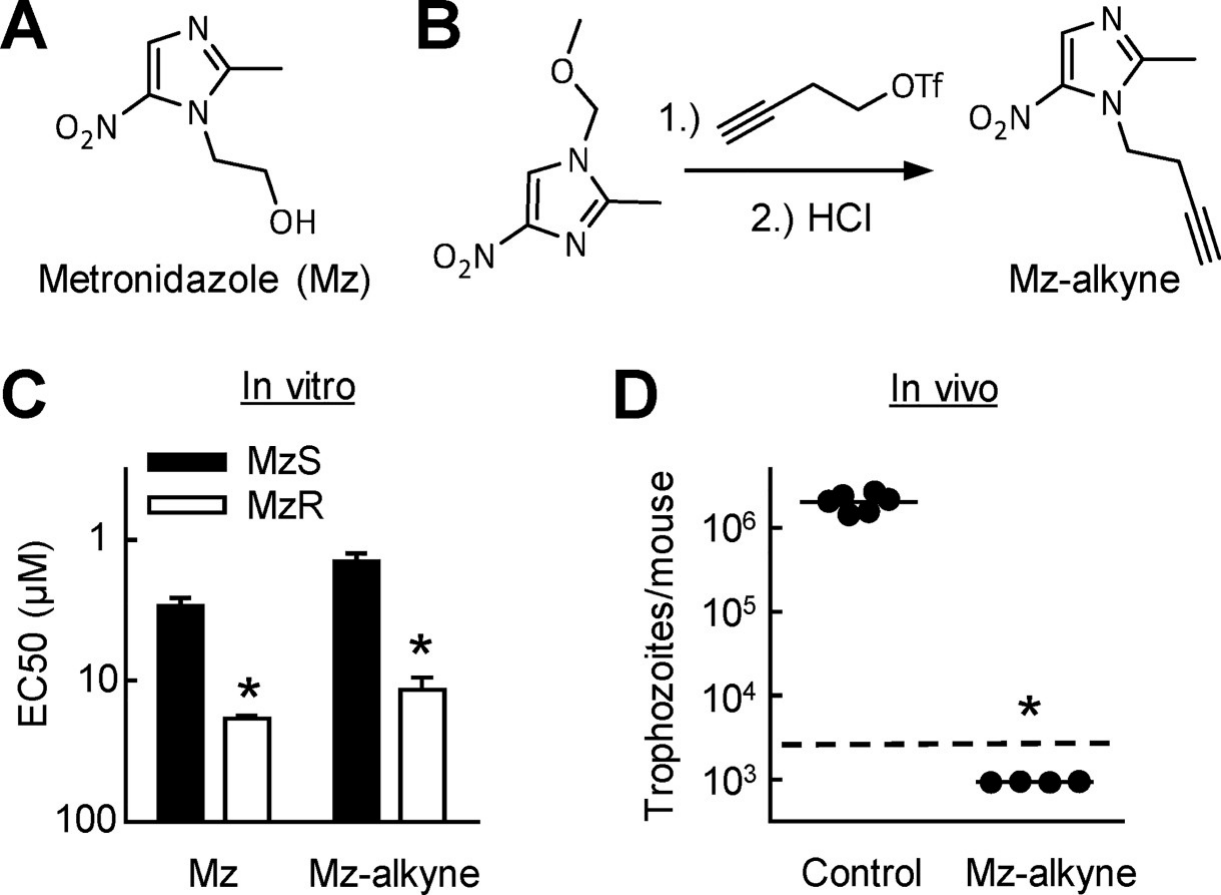
Synthesis and antigiardial activity of Mz-alkyne. A. Structure of metronidazole (Mz). B. Scheme of two-step synthesis of an alkyne-labeled derivative of Mz (Mz-alkyne). C. In vitro activity of Mz and Mz-alkyne against Mz-sensitive (MzS) and Mz-resistant (MzR) congenic strains of *G*. *lamblia* WB, as determined by EC50 assay. Results are shown as mean + SD (n = 3 independent experiments; *p<0.01 vs MzS cells). D. In vivo efficacy of Mz-alkyne against *G*. *lamblia* WB in a suckling mouse model of giardiasis. Starting one day after infection, mice were treated orally with a daily dose of 10 mg/kg Mz-alkyne for three days, or given PBS as a control, and trophozoites were enumerated in the small intestine. Each data point represents one animal; horizontal lines show the geometric means (*p<0.05 vs control). The dashed line depicts the assay sensitivity.

We first synthesized a Mz-like compound, Mz-alkyne, in which the hydroxyethyl group of Mz (Fig 2A) in the 1-position of the imidazole core was replaced with an ethyl acetylene group to introduce a terminal alkyne (Fig 2B). In vitro testing against the *G*. *lamblia* strain WB, which belongs to genetic assemblage A and was the first genome strain, in a 48 h growth and survival assay showed that Mz-alkyne was slightly more active than Mz (Fig 2C), which may be explained by its slightly greater predicted hydrophobicity, with a calculated logP value of 0.95 compared to -0.46 for Mz. Furthermore, both compounds were impacted by Mz resistance (MzR) to a similar degree, since a resistant *G*. *lamblia* line displayed a 5- to 10-fold increase in EC50 for both Mz and Mz-alkyne compared to the drug-sensitive parental line (Fig 2C). Mz-alkyne was also efficacious in vivo, because oral administration of a standard dose of 10 mg/kg over 3 days cleared intestinal *G*. *lamblia* infection in a suckling mouse model of giardiasis, which was employed to allow reliable infection with the WB strain (Fig 2D). These results show that Mz-alkyne is a potent 5-NI drug with in vitro and in vivo activities similar to or even greater than Mz.

### Detection of nitro drug-adducted proteins by click chemistry

**Fig 3:**
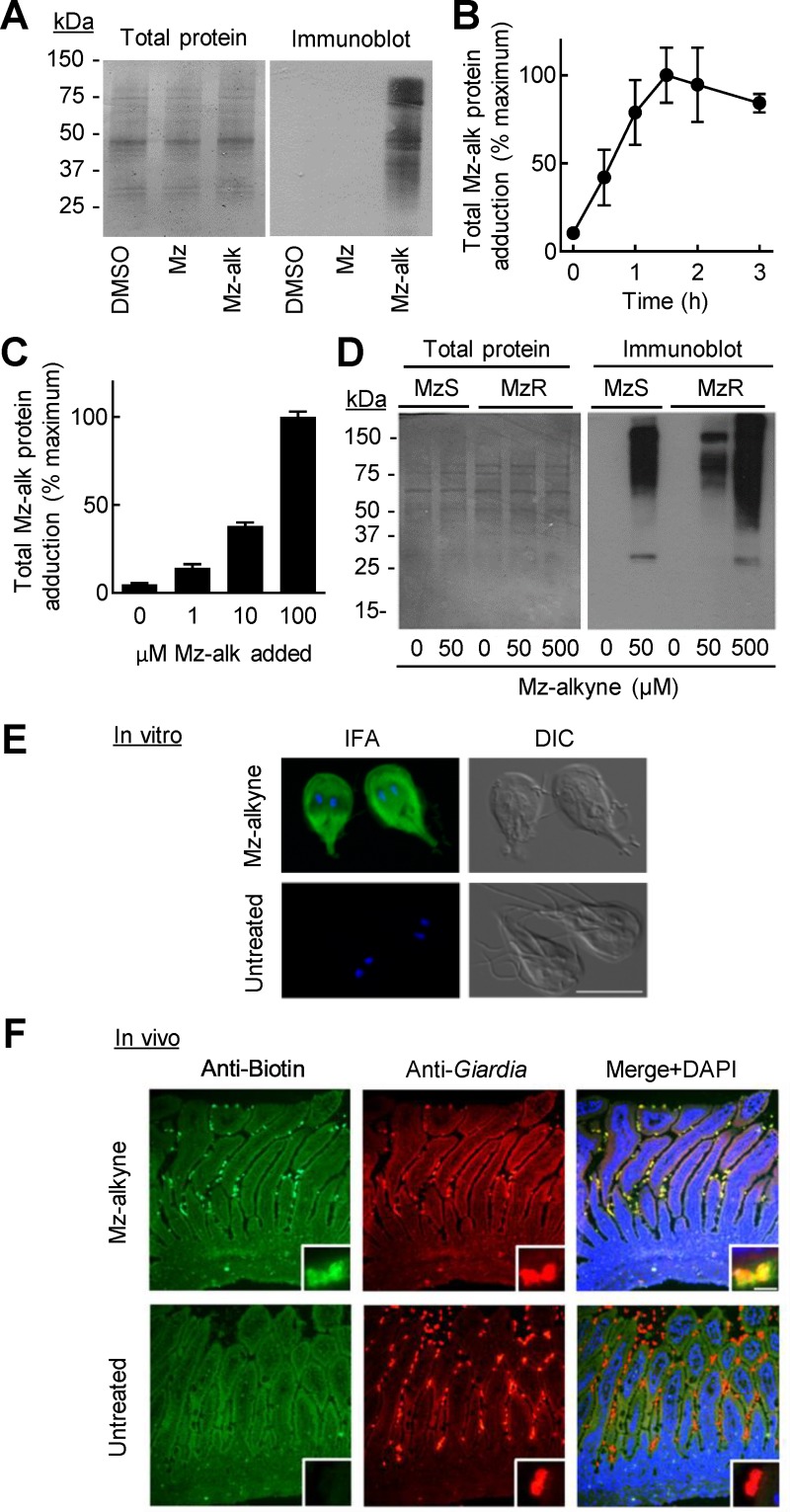
Biochemical detection of 5-NI drug adduction in *G*. *lamblia*. A. Trophozoites of *G*. *lamblia* WB were treated for 2 h with Mz-alkyne (Mz-alk) or Mz (both at ~30 x of their respective EC50), or with DMSO as a solvent control. After gel electrophoresis of cell extracts, adducted proteins were analyzed by immunoblotting using in situ click chemistry for coupling with azido-biotin followed by staining with HRP-labeled anti-biotin and visualization by chemiluminescence. Total protein was stained with Coomassie Blue. B,C. Trophozoites were treated with 100 μM (B) or the indicated concentrations (C) of Mz-alkyne for the indicated periods (B) or 2 h (C). Cells were washed and lysed, and the extracts were reacted with azido-biotin by the click reaction and coated onto 96-well plates. Bound biotin-labeled proteins were detected with streptavidin-HRP followed by incubation with the substrates TMB and H_2_O_2_. Data are mean ± SD (n = 3 replicates/group). D. Mz-sensitive (MzS) and congenic Mz-resistant (MzR) lines of *G*. *lamblia* WB were incubated for 2 h with the indicated concentrations of Mz-alkyne, and cell extracts were analyzed by immunoblotting for adducted proteins as done in panel A. E. Trophozoites attached to glass coverslips were treated for 15 min with 100 μM Mz-alkyne or left untreated, fixed with methanol, and permeabilized with Triton X-100. In situ click reaction was performed with azido-biotin followed by staining with Alexa 488-labeled anti-biotin (IFA, green), and counterstaining with DAPI (IFA, blue). Differential interference contrast (DIC) microscopy was used to visualize the entire cells (bar, 10 μm). F. Suckling mice infected with *G*. *lamblia* WB for 4 days were treated with 100 mg/kg of Mz-alkyne for 2 h or left untreated. Frozen sections of the small intestine were stained by in situ click chemistry with azido-biotin followed by Alexa 488-labeled anti-biotin (green). Trophozoites were detected with an antibody against a *G*. *lamblia*-specific protein, GASP-180 (red). DAPI was used for counterstaining (blue). Insets show higher magnifications of representative trophozoites.

We next tested the proposed identification strategy for 5-NI drug targets in cultures of *G*. *lamblia* trophozoites. As outlined in Fig 1, trophozoites were incubated with Mz-alkyne for different times and total cell extracts were prepared. The terminal alkyne groups of drug-adducted proteins were reacted with an azido-biotin using the click reaction, and the triazole-linked biotin conjugates were detected by immunoblotting and ELISA. Mz-alkyne treated *G*. *lamblia*, but not untreated or Mz-treated controls, showed multiple adducted bands by immunoblotting (Fig 3A). The band pattern did not parallel the total protein staining pattern, suggesting that protein adduction exhibited some selectivity. Moreover, ELISA measurements of protein adduction showed that adduction was detectable as early as 30 min after drug addition to the trophozoite cultures and increased in intensity in a time- and concentration-dependent manner, although adduction appeared to reach saturation by 1–2 h (Fig 3B and 3C).

To further explore the characteristics of the 5-nitro drug adduction targets under different conditions, we investigated whether resistance to Mz had an impact on adduction. MzR cells displayed fewer and less intense protein bands by immunoblotting of extracts compared to Mz-sensitive (MzS) cells when treated with equal drug concentrations (i.e., 10 x EC50 for MzS cells) of Mz-alkyne (Fig 3D). However, treatment of MzR cells with a 10-fold higher concentration (100 x EC50 in MzS cells, equivalent to ~10 x EC50 in MzR cells) of Mz-alkyne resulted in adduction levels similar to those in MzS cells treated with the standard drug concentration (10 x EC50 in MzS cells) (Fig 3D). These results demonstrate that multiple proteins are adducted even in MzR cells, although higher drug concentrations were required to achieve similar levels of adduction.

### Immunofluorescence analysis of adducted proteins in vitro and in vivo

Beyond facilitating biochemical target analysis, we asked if click chemistry-mediated 5-NI drug target recognition could be used for histological target identification. As a test, we detected adducted targets after incubation with Mz-alkyne by in situ click reaction with azido-biotin and staining of the biotin-labeled targets with a fluorochrome-labeled anti-biotin antibody. Mz-alkyne adducted proteins mainly localized diffusively in the cytoplasm of trophozoites (Fig 3E). Untreated controls showed no specific staining. Given the ready fluorescent detection of adducted targets in vitro, we also tested whether the click chemistry-based approach could visualize drug targeting in vivo. Suckling mice infected with *G*. *lamblia* WB were given a single dose of Mz-alkyne for 2 h or left untreated. Frozen sections of the small intestine were stained for drug-adducted proteins by in situ click reaction followed by fluorochrome-labeled anti-biotin, or for a *G*. *lamblia*-specific protein as a control. Immunofluorescence microscopy revealed that *G*. *lamblia* from Mz-alkyne treated mice stained strongly and selectively for adducted targets, while trophozoites from infected but untreated mice showed no specific staining (Fig 3F). Importantly, the Mz-alkyne treated mice had no specific staining signal in the surrounding intestinal tissue, clearly demonstrating the drug selectivity in vivo. Both groups of infected mice had the expected positive staining with the anti-*Giardia* antibody (Fig 3F). Taken together, these data demonstrate that our click chemistry-based strategy is a sensitive and specific method for detection of Mz-alkyne adducted proteins in *G*. *lamblia* in vitro and in vivo, and thus provided the functional justification for subsequent molecular identification of those targets.

### Mass spectrometric identification of Mz-alkyne adducted proteins

To apply the new target detection strategy to the identification of Mz-alkyne adducted proteins (the ‘adductome’), we treated *G*. *lamblia* WB trophozoites with a high but physiologically achievable Mz-alkyne concentration (100 μM; reported peak Mz plasma levels range from 70–230 μM), and affinity-purified the biotin-labeled (i.e., adducted) proteins from cell extracts using streptavidin affinity chromatography (as outlined in Fig 1) The purified proteins were then digested in situ with trypsin, and the released tryptic peptides were analyzed by LC-MS/MS. The identified mass spectra were aligned with tryptic peptides predicted from the genome sequence of *G*. *lamblia* WB (ATCC 50803) [42] to identify the Mz-alkyne adducted proteins. In control experiments, no proteins were identified with this method in solvent-treated cells, showing the excellent specificity of the assay.

The mass spectrometric analysis revealed a total of 161 unique proteins in *G*. *lamblia* that were adducted in three separate experiments with MzS *G*. *lamblia* (S1 Table). Of these, 51 showed the most consistent and significant adduction across all three experiments (Table 1), whereas the other 110 proteins were identified in only one or two of the three experiments or their variable spectral counts across the three experiments were not significant. The number of total adducted proteins represents 5–6% of the ~2,800 total proteins predicted to be expressed in the trophozoites of the parasite [43].

**Table 1:** Proteins adducted by Mz-alkyne in *G*. *lamblia*.

				Normalized spectral abundance factor x 100			
Gene ID (GL50803_)	Gene product	Size (aa)	Function	MzS cells (mean±SD)	MzR cells (mean)	Cell lysate (mean)	Potential as drug target
10311	Ornithine carbamoyltransferase	327	Metabolism	5.63±0.41	10.82	7.63	Yes [44]
14521	Peroxiredoxin 1	201	Redox	2.89±0.84	5.28	4.14	Unknown
5810	Hypothetical protein	131	-	2.69±0.76	0	2.12	Unknown
16453	Carbamate kinase	316	Metabolism	2.63±0.25	4.48	4.39	Yes [45,46]
112304	Elongation factor 1-alpha	442	Protein biosynthesis	2.51±0.57	4.80	3.77	Unknown
16076	Peroxiredoxin 1	201	Redox	2.49±0.50	5.28	4.14	Unknown
112103	Arginine deiminase	580	Metabolism	2.27±0.88	3.05	0.96	Yes [47,48]
6687	Glyceraldehyde 3-phosphate dehydrogenase	336	Metabolism	2.13±0.67	2.11	4.13	Unknown
103676	Alpha-tubulin	454	Cytoskeleton	2.01±0.67	3.90	3.05	Yes [45,49]
10429	Wos2 protein	185	Chaperone	1.83±0.50	0	0	Unknown
7110	Ubiquitin	82	Proteasome	1.79±0.41	0	0	Unknown
14614	Eukaryotic translation initiation factor 5A	149	Protein biosynthesis	1.78±0.29	2.38	0	Unknown
3910	Hypothetical protein	123	-	1.74±0.46	0	0	Unknown
6430	14-3-3 protein	248	Signaling	1.51±0.54	2.85	0	Unknown
17153	Alpha-11 giardin	307	Cytoskeleton	1.41±0.54	2.31	1.81	Unknown
21942	NADP-specific glutamate dehydrogenase	449	Metabolism	1.41±0.66	0.79	1.85	Unknown
17163	Peptidyl-prolyl cis-trans isomerase B precursor	168	Protein folding	1.36±0.62	2.11	1.65	Unknown
17547	Ribosomal protein L4	316	Protein biosynthesis	1.21±0.22	0	1.76	Unknown
88765	Cytosolic HSP70	664	Chaperone	1.12±0.37	2.13	0.84	Unknown
15869	GTP-binding nuclear protein RAN/TC4	226	Signaling	1.11±0.40	1.57	0	Unknown
17327	Xaa-Pro dipeptidase	444	Proteasome	1.03±0.23	1.59	1.87	Unknown
11118	Enolase	445	Metabolism	1.03±0.22	0	0	Unknown
9909	Pyruvate, phosphate dikinase	884	Metabolism	1.03 ±0.42	0.80	1.88	Unknown
21628	Hypothetical protein	383	-	1.02±0.28	0	1.45	Unknown
9779	UPL-1	310	Metabolism	0.97±0.11	0	0.89	Unknown
15520	Ribosomal protein L21	159	Protein biosynthesis	0.97±0.42	2.23	3.49	Unknown
17244	Ribosomal protein L7a	225	Protein biosynthesis	0.95±0.25	0	0	Unknown
90872	Phosphoglycerate kinase	409	Metabolism	0.92±0.48	0	0.68	Unknown
19436	Ribosomal protein L7	235	Protein biosynthesis	0.91±0.24	0	0	Unknown
17054	Acidic ribosomal protein P0	326	Protein biosynthesis	0.88±0.47	1.09	0	Unknown
10255	Translation initiation factor eIF-4A, putative	391	Protein biosynthesis	0.85±0.06	0.91	0	Unknown
17060	Protein 21.1	623	Cytoskeleton	0.82±0.22	0.57	0.89	Unknown
13864	Heat shock protein HSP 90-alpha	324	Chaperone	0.77±0.28	1.09	0.86	Yes [50]
16431	Ribosomal protein L19	196	Protein biosynthesis	0.75±0.17	0	0	Unknown
11654	Alpha-1 giardin	295	Cytoskeleton	0.75±0.17	1.20	2.82	Unknown
17121	Bip	677	Chaperone	0.73±0.25	0	0	Unknown
16525	Ribosomal protein L3	379	Protein biosynthesis	0.64±0.27	0	0.73	Unknown
7766	Ribosomal protein SA	245	Protein biosynthesis	0.63±0.27	1.44	0	Unknown
12102	Elongation factor 1-gamma	402	Protein biosynthesis	0.60±0.27	0.88	0.69	Unknown
13747	C4 group specific protein	198	-	0.60±0.17	0	0	Unknown
13561	Translation elongation factor	220	Protein biosynthesis	0.53±.15	1.61	0	Unknown
8118	Ribosomal protein S2	242	Protein biosynthesis	0.49±0.13	0	0	Unknown
16086	Ribosomal protein L2	251	Protein biosynthesis	0.47±0.13	0	0	Unknown
17400	Hypothetical protein	314	Hypothetical	0.47±0.11	0	0	Unknown
11043	Fructose-bisphosphate aldolase	323	Metabolism	0.36±0.11	1.10	0	Unknown
93358	Alcohol dehydrogenase	888	Metabolism	0.36±0.16	1.20	0.31	Yes [51]
11390	Kinase, NEK	777	Signaling	0.33±0.17	0.46	0	Unknown
15832	Aminoacyl-histidine dipeptidase	524	Proteasome	0.29±0.13	0	0	Unknown
13500	TCP-1 chaperonin subunit theta	563	Chaperone	0.26±0.06	0	0	Unknown
86511	Acyl-CoA synthetase	970	Metabolism	0.21±0.12	0.36	0.29	Unknown
17063	Pyruvate-flavodoxin oxidoreductase	1199	Redox	0.10±0.03	0	0.46	Unknown

Trophozoites of Mz-sensitive (MzS, experiments 1–3) and congenic Mz-resistant (MzR) lines of *G*. *lamblia* WB (GL50803) were treated with Mz-alkyne for 2 h, after which cell lysates were prepared and reacted with azido-biotin using the click reaction. In a separate experiment, cell lysates were prepared from untreated *G*. *lamblia* WB trophozoites, and incubated with Mz-alkyne and dithionite as an external reducing system before reacting with azido-biotin by the click reaction. Biotin-labeled proteins were purified by streptavidin affinity chromatography, and identified by in situ trypsin digestion and subsequent LC-MS/MS analysis. Peptide spectral counts were tabulated, and used to calculate the normalized spectral abundance factor (NSAF), which is shown as mean ± SD of three experiments with MzS cells, or as means for MzR cells and cell lysates. The entries represent the adducted gene products whose NSAF were significantly different (p<0.05) from no adduction. The complete list of all identified gene products is shown in S1 Table.

**Fig 4:**
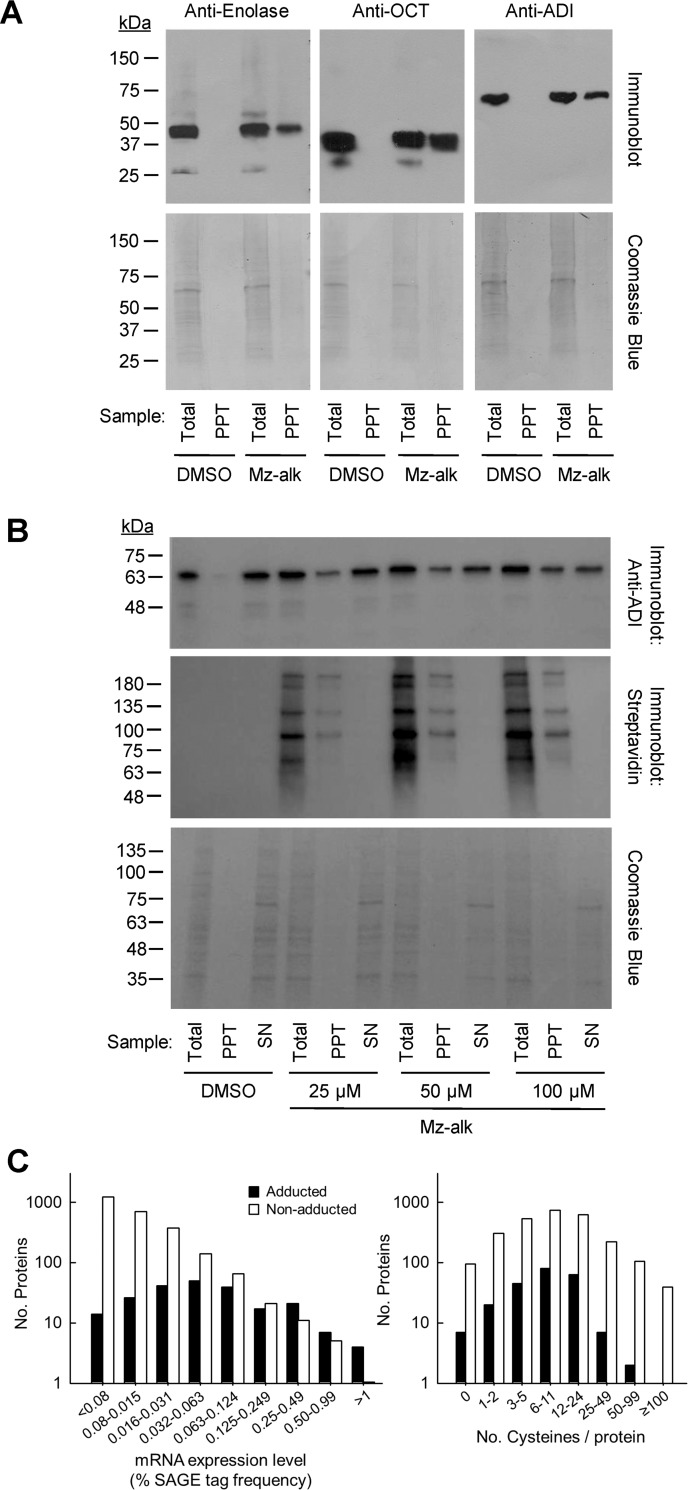
Confirmation of nitro drug adduction and target protein characteristics. Trophozoites of *G*. *lamblia* WB were treated for 2 h with 100 μM (A) or the indicated concentrations (B) of Mz-alkyne (Mz-alk) or solvent (DMSO) alone, and cell lysates were prepared and reacted with azido-biotin using the click reaction. Biotin-labeled proteins were purified by streptavidin affinity chromatography, and identified by in situ trypsin digestion and subsequent LC-MS/MS analysis. A, B. Whole cell extracts (Total), streptavidin affinity-precipitated proteins (PPT), and the remaining supernatants after precipitation (SN) were analyzed by immunoblotting with specific antibodies against enolase, OCT, or ADI. Total protein was detected by Coomassie staining. To control for the efficiency of affinity precipitation, immunoblots were stained in parallel with HRP-conjugated streptavidin. C. Adducted proteins (closed bars) and all others (non-adducted) proteins (open bars) predicted from the *G*. *lamblia* WB genome were analyzed for expression levels, as determined by SAGE tags in prior work [43] (left panel), or cysteine content (right panel).

For three examples, OCT, enolase, and ADI, we confirmed by immunoblotting with specific antibodies that these proteins were labeled with biotin and thus adducted by Mz-alkyne (Fig 4A). Further quantitative analysis of one of these enzymes, ADI, showed that the non-adducted fraction of the enzyme decreased in a concentration-dependent manner from 80% at 25 μM Mz-alkyne to 74% at 50 μM and 55% at 100 μM as determined by scanning densitometry of immunoblots, although even at the highest tested drug concentration close to half of the ADI remained non-adducted (Fig 4B).

Furthermore, proteomic analysis of MzR cells, when treated with Mz-alkyne at a concentration that was functionally equivalent to the one used in MzS cells, revealed 49 unique proteins (Table 1 and S1 Table). This number was ~3-fold lower than the total in MzS cells, which is consistent with the ~3-fold lower total spectral counts in this experiment (S1 Table). Importantly, all of the adducted proteins in MzR cells were also identified in MzS cells, indicating that drug adduction in MzR cells was qualitatively similar to MzS cells, albeit perhaps less efficient, suggesting that drug resistance is related to decreased efficiency of target adduction, but not changes in the spectrum of target proteins.

To further test this notion, we performed drug adduction assays with *G*. *lamblia* trophozoite lysates under cell-free conditions with an external reducing system (dithionite) to activate the Mz-alkyne. The spectrum of adducted proteins in drug-exposed extracts from MzS cells was found to be overlapping with the adducted proteins in drug-treated live MzS and MzR cells, since all of the 56 adducted proteins that we identified in cell lysates were also found in live cells (Table 1 and S1 Table). These results indicate that the target specificity of protein adduction is largely independent of the mechanism of Mz-alkyne activation. Together, our findings suggest that one or several of the 161 adducted proteins in MzS cells are 5-NI drug targets that could mediate the antigiardial actions of Mz-alkyne, and that the majority of these targets are identical in MzR cells and may be exploitable for drug development to overcome Mz resistance.

Comparison of the functional classes (e.g. ribosomal proteins, energy metabolism, cytoskeleton, protein kinases) represented among all the adducted proteins and the total predicted proteome of *G*. *lamblia* did not reveal enrichment of any particular class (S1 Table). Evaluation of the relative abundance of adducted proteins was more informative. For example, the metabolic enzymes, OCT, ADI, carbamate kinase, and glyceraldehyde-3-phosphate dehydrogenase, were among the top ten proteins with the highest NSAF (Table 1), and their corresponding mRNAs were previously shown to be abundant in trophozoites [52], suggesting that high gene expression, and thus presumably target abundance, is a predictor for adduction. Consistent with this notion, drug adduction generally correlated with mRNA levels, at least for genes with medium to high expression (Fig 4C). In contrast, protein adduction was largely independent of the number of cysteine residues, which had previously been shown to be primary targets of activated 5-NIs [53], since the percentage of adducted compared to non-adducted proteins was similar for all proteins with <50 cysteines (Fig 4C). Interestingly, proteins very rich in cysteines (>50 residues) were not adducted at the expected frequency, perhaps because most of the cysteine-rich proteins are variant-specific proteins (VSPs) whose cysteine residues form disulfide bonds that may prevent adduction. VSPs are also expressed on the surface of trophozoites where they might be less accessible to activated 5-NI intermediates, although we found adduction of other proteins with surface localization, such as α1-giardin [54].

### Structural basis of drug adduction-induced ADI inhibition

**Fig 5:**
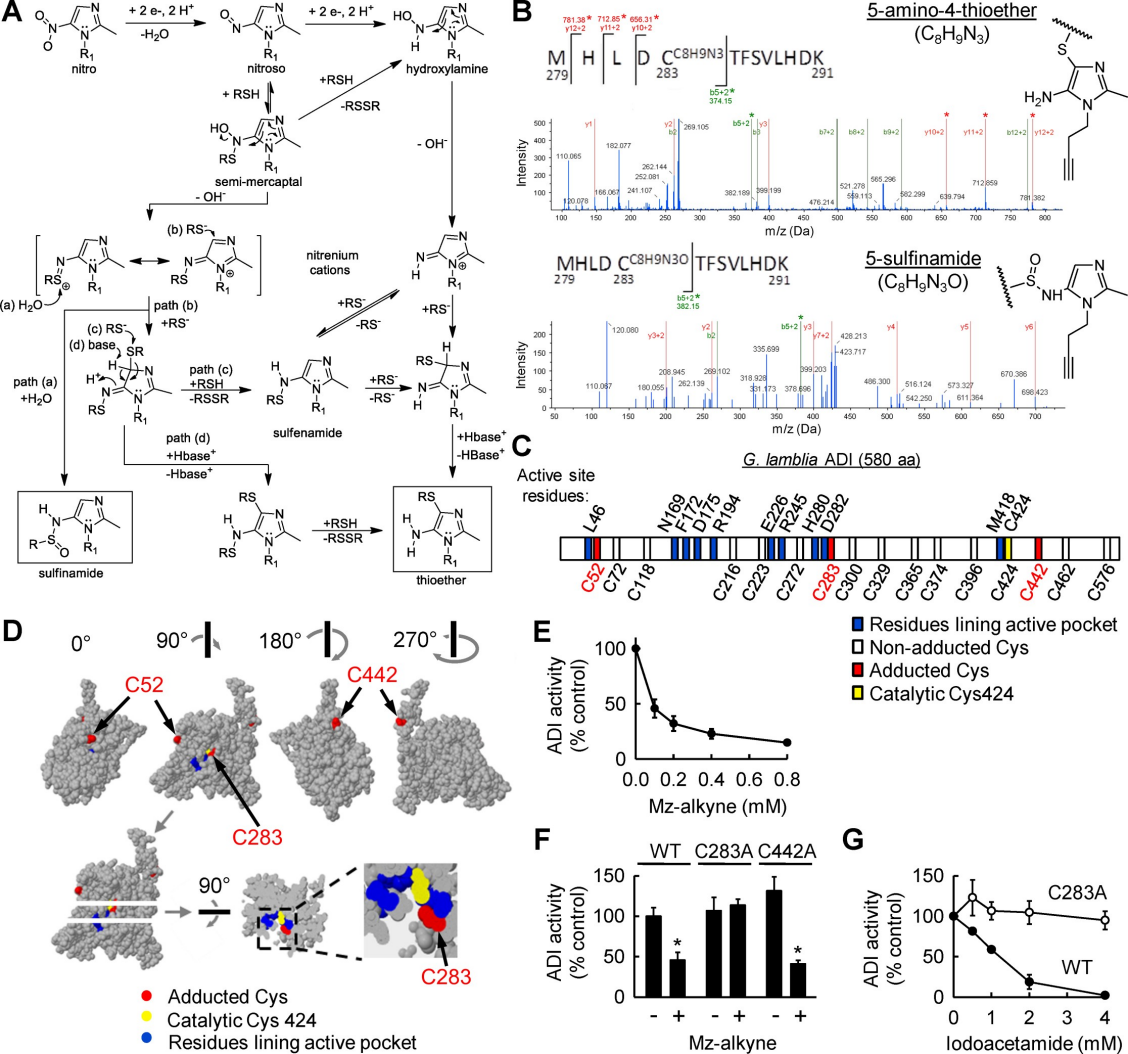
Functional impact of 5-NI adduction of ADI. (A) Proposed reaction mechanisms and structures of Mz-alkyne adducts on protein targets. (B) Example MS/MS spectra of the ADI peptide, 279-MHLDCTFSVLHDK-291, obtained by tryptic digestion of Mz-alkyne adducted ADI-HA. On cysteine 283, three y+2 ions and one b+2 ion with an added mass of 147 were observed that constitute evidence for 5-amino-4-thioether (C8H9N3) adduction, and one b+2 ion with an added mass of 163 that provides evidence for 5-sulfinamide (C8H9N3O) adduction. The predicted structures of these cysteine adducts are shown on the right. (C) Scheme of *G*. *lamblia* ADI with known active site residues labeled above and cysteines labeled below. (D) Homology model of ADI protein structure and adduction sites. Residues lining the active site pocket are shown in blue, the catalytic cysteine 424 in yellow, and adducted cysteines in red. Images on top show the whole protein from different angles. One of the images was optically sectioned (dashed arrows) and rotated 90° around the indicated horizontal axis to reveal a top view of the section. The inset shows a magnification of the active site pocket. The adducted cysteine 283 is located at the entrance to the pocket. (E) ADI activity in lysates of *G*. *lamblia* trophozoites treated with the indicated concentrations of Mz-alkyne (mean ± SD, n = 3 experiments). (F) Activity of recombinant ADI produced as wild-type (WT) or with mutations of cysteine 283 to alanine (C283A) or cysteine 442 to alanine (C442A) after 2 h treatment with (+) or without (-) Mz-alkyne under cell-free conditions in the presence of dithionite. (G) Activity of WT and mutant (C283A) ADI after 2 h treatment with iodoacetamide. Activities in G and H are expressed relative to solvent controls (mean +/- SD, n = 3; *p<0.05 vs controls).

To determine whether 5-NI drug adduction of the identified protein targets has functional consequences, we focused on ADI as an example of an enzyme with critical functions in *G*. *lamblia* metabolism [48]. As a first step, we sought to define the amino acid residues that are adducted by Mz-alkyne. To employ a mass spectrometry strategy, we first needed to consider plausible adduct reaction mechanisms and structures (Fig 5A). Because most nitrosoimidazoles, the most immediate products of the initial 2 e- reduction of the 5-NI prodrug, are highly reactive, the proposed pathways are based on those observed with the less reactive nitrosobenzene derivatives for which extensive mechanistic studies have been reported [55]. Two plausible adduct structures, namely sulfinamide and amino-thioether, could predictably result from the nitrosoimidazole formed upon reduction of Mz-alkyne (Fig 5A). Kinetic studies of nitrosobenzenes after thiol addition indicate the rapid equilibrium formation of an intermediate, probably a semi-mercaptal, which decays in a slower, irreversible reaction to give the final products [56,57]. The rate-determining step for the second reaction is probably the formation of a nitrenium-type cation formed by loss of hydroxide from the semi-mercaptal and stabilized by resonance with S [53]. This reaction would be expected to be much faster with the imidazoles than the benzenes due to the extra “push” given by the imidazole N as shown by the arrows in the semi-mercaptal structure (Fig 5A). The nitrenium cation is highly electrophilic and will react rapidly with available nucleophiles. With low thiol concentrations, the principle product with nitrosobenzenes is the sulfinamide [57,58] formed by attack of water on the S (path a in Fig 5A) [56]. With excess thiol present, sulfenamides [58] and amino-thioethers [59] can also be produced. Both of these products can arise via thiol addition to the imidazole ring of the nitrenium cation (path b in Fig 5A). The resulting dithiolated intermediate can then re-aromatize by thiolytic cleavage to give a sulfenamide (path c in Fig 5A), which can then rearrange by several pathways to give an amino-thioether [57]. Alternatively, re-aromatization can be accomplished by base catalysis (path d in Fig 5A) to give a thioether sulfenamide. Thiolytic cleavage of the more labile S-N bond of this species also leads to an amino-thioether. An alternative, more direct pathway to the amino-thioether starts with the hydroxylamine (top right side in Fig 5A), formed either by further 2 e- reduction of the nitrosoimidazole or thiolytic cleavage of the semi-mercaptal. The imidazole hydroxylamines are again more reactive than the corresponding benzenes, but, unlike the nitrosoimidazoles, they are generally stable enough to be characterized directly [60,61]. These studies indicate that the thioether is the major product in the presence of thiol [60], which is consistent with the slow step being loss of hydroxide and formation of a reactive nitrenium cation, analogous to the reaction of the semi-mercaptal. Again, this reaction is predictably much faster with the imidazoles than the benzene hydroxylamines. Addition of thiol to the nitrenium cation followed by base-catalyzed re-aromatization yields the thioether (bottom right in Fig 5A). Alternatively, the nitrenium cation could first form the sulfenamide, followed by rearrangement to the thioether [61].

Armed with plausible adduct structures, we next constructed an expression vector for an epitope (hemagglutinin peptide, HA)-tagged form of ADI to facilitate target purification and detailed mass spectrometric analysis. *G*. *lamblia* trophozoites were stably transfected with the ADI expression vector and treated with Mz-alkyne. ADI-HA was purified from extracts by affinity purification with anti-HA coupled sepharose and digested in situ with trypsin, and the resulting peptides were analyzed by LC-MS/MS. We then tested ADI-HA for the two adduct structures discussed above, the 5-amino-4-thioether (C8H9N3) and 5-sulfinamide (C8H9N3O). In addition, we searched for other potential adduct structures, including C8H10N3O, C8H8N3O, C8H7N3O, C8H7N2O, C8H10N2, C8H8N2, and C8H7N2, which are theoretically possible although little published evidence exists in support of their stable existence. With 100% coverage of all predicted tryptic peptides, we found that Mz-alkyne adducted several but not all of the 16 cysteines in ADI, as evidenced by the occurrence of multiple b- and y-ions consistent with C8H9N3 adducts on cysteines 52, 283, and 442 (Fig 5B and 5C). We obtained additional but more limited proof (one ion each) for C8H9N3O adducts on cysteines 52 and 283 (Fig 5B and 5C). No evidence was found for adduction of the catalytic cysteine 424 [47] or any of the other postulated adduct structures. These results show that both predicted adducts, 5-amino-4-thioether and 5-sulfinamide, are found on a limited number of cysteines in ADI, while the majority of cysteines were not adducted (Fig 5C).

To help interpret the adduct findings, we sought to locate the adducted cysteines in the three-dimensional structure of ADI. Based on the crystal structure of a related enzyme, *Pseudomonas aeruginosa* ADI (24% amino acid identity and 42% positives) [39], we employed molecular homology modeling to predict the location of the adduction sites in *G*. *lamblia* ADI, particularly in relation to the previously mapped active site pocket [47]. The resulting model predicts that the catalytic cysteine 424 is located deep in the active site pocket, while cysteine 283 is located at the entrance to that pocket, and cysteines 52 and 442 are on the protein surface away from the active site (Fig 5D). Thus, while we found no adduction of the catalytic cysteine 424, the modeling suggests that adduction on cysteine 283 may impair catalytic activity by interfering with substrate access and/or active site conformation.

To begin to test this hypothesis, we determined the effect of Mz-alkyne on ADI activity in live *G*. *lamblia*. The drug inhibited ADI in a concentration-dependent fashion by >80% relative to untreated cells (Fig 5E), but had no impact on total ADI levels (Fig 4A and 4B). The residual ADI activity measured in drug-treated cells may be related to incomplete adduction of the enzyme, since ~50% of ADI remained non-adducted at 100 μM Mz-alkyne (Fig 4B), which corresponded closely to the degree of ADI enzyme inhibition at the same drug concentration (Fig 5E). For further exploration of the mechanisms of ADI inhibition, we employed targeted mutagenesis and a cell-free assay to exclude other cellular factors that might affect ADI activity. Recombinant His-tagged ADI was produced in wild-type form or with mutations of cysteines 283 or 442 to alanine, which is structurally similar to cysteine but lacks the reactive sulfhydryl group. Purified enzymes were incubated with and without Mz-alkyne in the presence of dithionite as an external reducing and activating agent, and then tested for enzymatic activity. Wild-type ADI was significantly inhibited by activated Mz-alkyne under cell-free conditions (Fig 5F), thus paralleling the findings in live cells treated with the drug. In contrast, the C283A mutant was resistant to inhibition by Mz-alkyne, whereas the C442A mutant of ADI was inhibited as much as the wild-type enzyme (Fig 5F). These findings indicate that cysteine 283 is critical for mediating the adduction-induced ADI inhibition, while the surface-located cysteine 442 had no functional significance for drug-induced enzyme inhibition. This conclusion was further supported by the observation that a different thiol-modifying agent, iodoacetamide, which we confirmed to bind covalently to cysteine 283, also inhibited wild-type ADI, but not the C283A mutant, in a concentration-dependent fashion (Fig 5G).

### Nitro drug-adducted proteins as new drug targets

**Fig 6:**
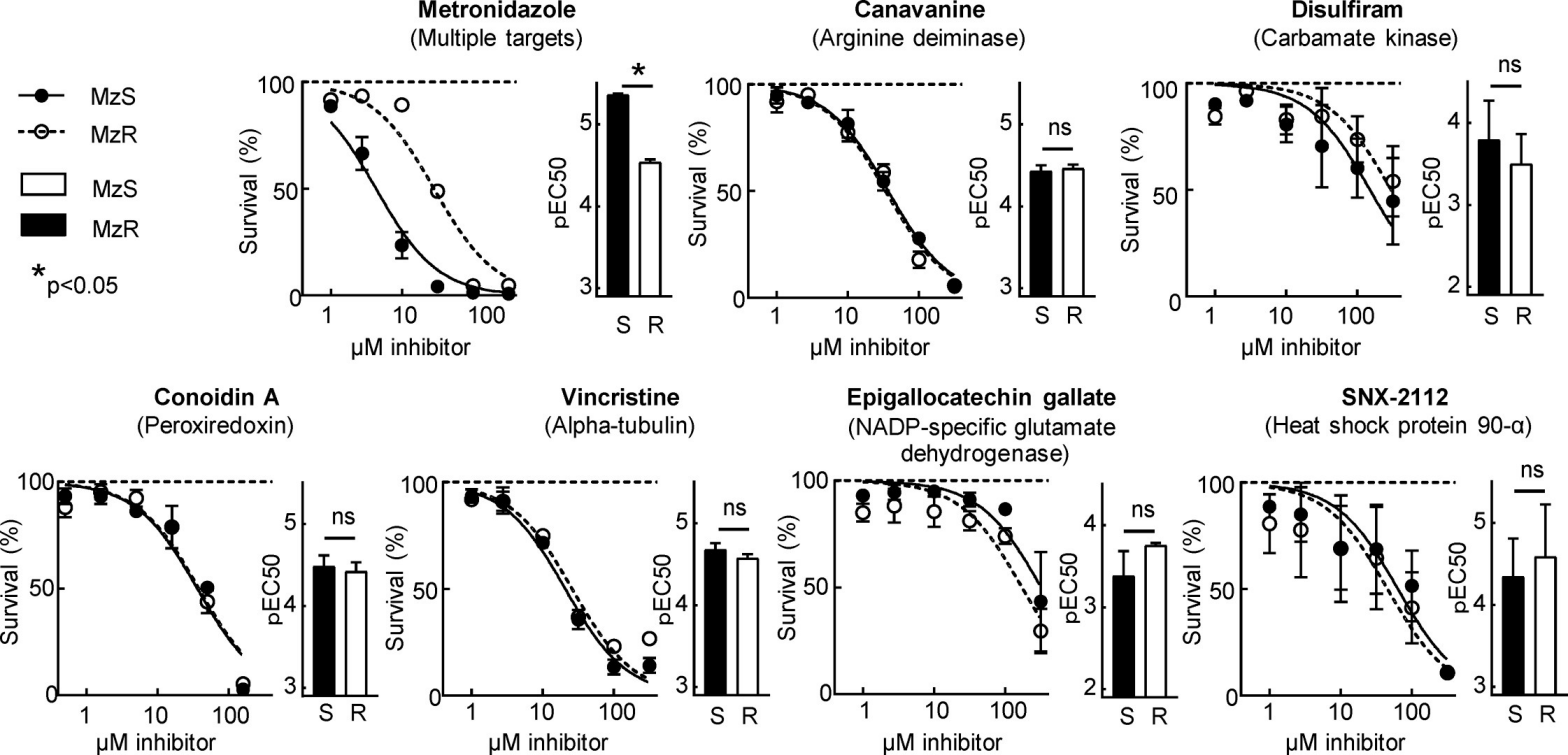
Therapeutic potential of 5-NI drug targets. A MzS and congenic MzR line of *G*. *lamblia* WB were tested for susceptibility to the indicated inhibitors of the targets identified by adductomics. The line graphs show concentration-response curves for parasite survival after treatment with the indicated inhibitor concentrations (means ± SE, n = 3–4; horizontal lines represent parasite numbers untreated controls). The bar graphs depict the pEC50 values derived from the concentration-response curves (means + SE, n = 3–4; *p<0.05 vs MzS cells,; ns, not significant).

A literature review of the 30 proteins found to be adducted by Mz-alkyne in both MzS and MzR cells revealed that at least six of them had previously been explored as drug targets in *G*. *lamblia* (Table 1), suggesting that the 5-NI adductome was enriched for potential drug targets. However, none of these targets had been investigated for their utility as alternative drug targets for overcoming Mz resistance. Therefore, we selected four known targets (ADI, α-tubulin, carbamate kinase, heat shock protein 90-α) and two new targets, NADP-specific glutamate dehydrogenase (GDH) and peroxiredoxin, and determined the antigiardial activity of inhibitors of these targets. Inhibition of all six targets led to effective trophozoite killing, with similar pEC50 values in MzS and MzR strains of *G*. *lamblia* (Fig 6). In contrast, Mz showed the expected marked difference in pEC50 values (equivalent to a 6-fold difference in EC50 values) between sensitive and resistant cells. These results demonstrate that proteins identified in the 5-NI drug adductome can be exploited as new drug targets for overcoming nitro drug resistance in *G*. *lamblia*.

**Fig 7:**
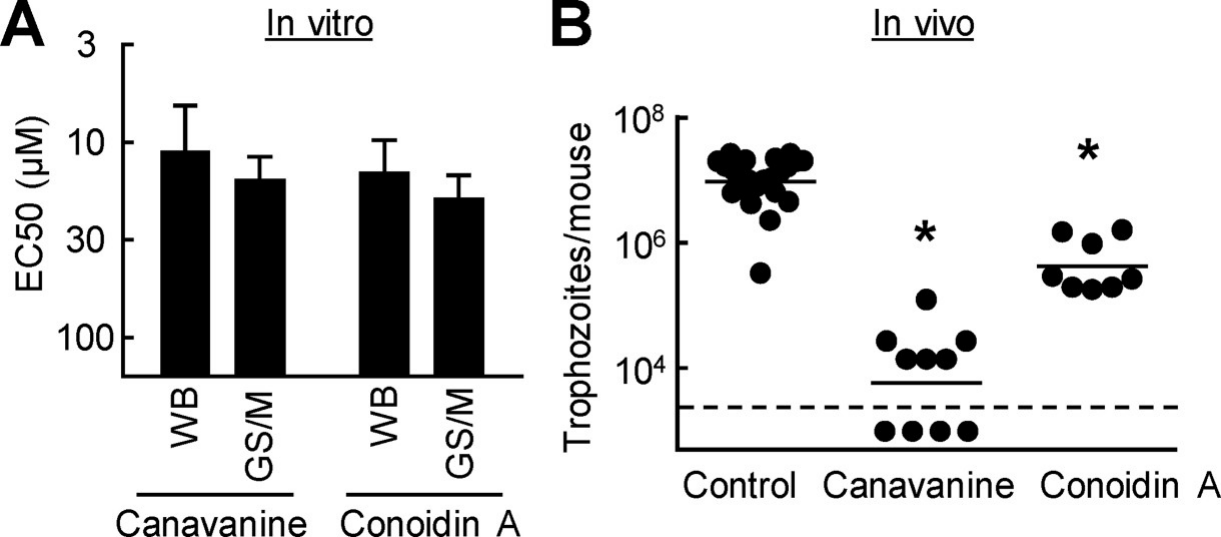
In vivo efficacy of inhibitors of 5-NI targets against giardiasis. A. In vitro activity of the ADI inhibitor, canavanine, and the peroxiredoxin inhibitor, conoidin A, against *G*. *lamblia* GS/M, as determined by EC50 assay. Results are mean + SD (n = 3 independent experiments). B. Adult mice infected with *G*. *lamblia* GS/M were treated orally with canavanine (2 g/kg) for a total of three doses, or with conoidin A (100 mg/kg) for a total of four doses, or given vehicle alone (Control), over a 3-day period. On day 5, live trophozoites were enumerated in the small intestine. Each data point represents one animal, horizontal bars show geometric means (*p<0.05 vs controls). The dashed horizontal line represents the assay sensitivity.

To further test the potential therapeutic utility of the identified drug targets, we selected two representative targets and their inhibitors, the ADI inhibitor canavanine and the peroxiredoxin inhibitor conoidin A, and evaluated them for in vitro cytotoxicity and in vivo efficacy. Testing in human HeLa cells revealed pCC50 values of 4.08 ± 0.19 and 5.87 ± 0.07 for canavanine and conoidin A, respectively (mean ± SE; n = 4–5). Together with the findings on giardicidal potency (Fig 6), these data show that canavanine had modest selectivity for the parasite (selectivity index = 2.5), while conoidin A had no selectivity (selectivity index, 0.04). For in vivo efficacy tests and to expand the findings to a divergent strain of *G*. *lamblia*, we first confirmed that the two inhibitors were active against the GS/M strain of *G*. *lamblia*, which belongs to genetic assemblage B and can readily infect adult mice (Fig 7A). Subsequently, we infected adult mice with this *G*. *lamblia* strain and treated them daily by oral gavage of canavanine or conoidin A, or solvent as a control. Both inhibitors significantly reduced infection by 10- to 50-fold compared to controls (Fig 7B). These data suggest that the respective targets of the two inhibitors, ADI and peroxiredoxin, have therapeutic potential in vivo.

## Discussion

Nitro-heterocyclic drugs are potent antimicrobials with a wide range of target pathogens, but the details of their mechanism-of-action remain incompletely understood. It is generally accepted that they are prodrugs which are converted to reactive intermediates by reduction in the target microbes. The intermediates form covalent adducts with proteins or DNA, leading to toxic cell damage, but the specific targets and molecular mechanisms that are directly responsible for cell death in different microbes are not well defined [6,10]. This uncertainty might be one of the reasons why commercial nitro drug development has largely ceased, as poorly defined or numerous targets present unique problems for drug design and optimization. In addition, approved nitro drugs can have significant adverse effects, including neurologic maladies (peripheral neuropathy, cerebellar syndrome, encephalopathy, and meningitis) [62,63], and intolerable nausea and gastric cramping [64]. However, the generally excellent potency and utility of nitro drugs suggest that it should be possible to leverage molecular identification of those targets for new drug design by providing an enriched list of potential targets with a likely if not proven role in mediating the actions of an already successful drug class, while at the same time potentially avoiding the liabilities of that class. Our study provides strong support for this hypothesis, because several of the proteins we identified in the 5-NI drug adductome had already been explored as potential drug targets [44–49] and we showed that two of them are valid new targets in *G*. *lamblia*. Importantly, our adductome findings revealed that most of the adducted proteins remain available as targets even in nitro drug-resistant cells. Testing of several inhibitors of these targets confirmed that resistance had no impact on their antigiardial activity. Therefore, our strategy of combining click chemistry with proteomics provides a novel approach for identifying drug targets in *G*. *lamblia* that can be exploited for the development of new classes of antimicrobial drugs.

Two of the identified 5-NI-adducted proteins, GDH and peroxiredoxin, represent new drug targets for treatment of giardiasis. GDH catalyzes the reversible conversion of glutamate to α-ketoglutarate and ammonia while reducing NADP^+^ to NADPH. While in most eukaryotic and prokaryotic organisms, the main role of NADP-dependent GDH is to synthesize glutamate for protein synthesis, *Giardia* uses this enzyme, in cooperation with alanine aminotransferase, to convert pyruvate to alanine under low-oxygen conditions [65]. *G*. *lamblia* possesses a single, highly expressed *Gdh* gene that encodes for a protein with NADPH-dependent GDH activity [66]. The gene is highly conserved among different *G*. *lamblia* isolates, suggesting it mediates essential parasite functions, yet has only limited homology (29% amino acid identity) with the two human GDH isoenzymes [67]. Together, these observations suggest that GDH has excellent potential as a new antigiardial drug target, a notion borne out by our initial observations with a broad inhibitor of this enzyme.

Another nitro drug target, peroxiredoxin, had also not been explored before as a potential drug target in *G*. *lamblia*. The parasite possesses two closely related peroxiredoxins, Prx1a and Prx1b, which detoxify hydrogen peroxide and alkyl-hydroperoxides [68]. They are upregulated by oxidative stress and contact with intestinal epithelial cells, suggesting they are active during host infection [52]. In cooperation with thioredoxin and thioredoxin reductase, peroxiredoxins are presumed to be central to antioxidant defense in *G*. *lamblia*, which otherwise lacks many of the conventional antioxidants such as catalase, glutathione peroxidase and superoxide dismutase [69]. We found that inhibition of peroxiredoxin is toxic to *G*. *lamblia* in vitro and in vivo, underlining the essential nature of this antioxidant system for parasite survival. As for the other targets identified in the 5-NI drug adductome, the giardicidal activity of the peroxiredoxin inhibitor was not affected by 5-NI drug resistance. Although the commercially available inhibitor, conoidin A, showed only modest antigiardial potency, the parasite enzymes have only 50–53% amino acid identity with the most closely related human peroxiredoxins, suggesting that development of a more potent and selective inhibitor should be feasible.

In addition to identifying new drug targets, our studies confirm and extend the value of several previously explored drug targets. For example, we observed that ADI is adducted by nitro drugs. Consistent with this finding, another study found that ADI was adducted by a different nitro-heterocyclic compound [14]. This enzyme is important in the energy metabolism of several anaerobes, including *G*. *lamblia*, as it converts arginine to citrulline, ultimately leading to ATP production [38]. In addition, it may contribute to evasion of innate and adaptive immune defenses by competing for arginine with epithelial nitric oxide synthase [70], and thereby attenuating production of nitric oxide as a potential antigiardial effector, and by converting peptidyl arginine to peptidyl citrulline, thus potentially altering the antigenicity of surface proteins [48]. Because of its importance for *G*. *lamblia*, its immunomodulatory functions and its absence in human cells, the present and independent prior studies on the enzyme suggested that ADI is a potential drug target [47]. Our inhibition experiments with an arginine analog, canavanine, suggest that ADI blockade can indeed be a potent strategy to kill *G*. *lamblia* independent of 5-NI drug resistance. It is possible that canavanine could be incorporated into proteins during protein synthesis, which may result in non-functional or incorrectly folded proteins. However, our cytotoxicity assays showed that the inhibitor has modest selectivity for the parasite over human cells, suggesting that ADI inhibition may be the more important mechanism-of-action against *G*. *lamblia*. Consistent with this notion, previous genetic ADI knock-down approaches also indicated that ADI inhibition is lethal for the parasite [47]. Two other enzymes involved in arginine metabolism, OCT and carbamate kinase, were also found to be adducted by 5-NI drugs in our work, and OCT was previously shown to be adducted by another nitro-heterocyclic compound [14]. These enzymes, like ADI, have only limited or minimal homology to human proteins. Because *G*. *lamblia* utilize arginine as a primary energy source [71], our data suggest that targeting enzymes involved in arginine-dependent energy generation may be a powerful strategy for creating a new class of antigiardial agents.

The critical targets that mediate the actions of nitro drugs are incompletely understood. Irreparable DNA damage is important in bacteria [12] and DNA damage also occurs in protozoa [13], but the importance of this mechanism relative to others remains to be shown in *Giardia* [13]. For example, nitro drugs can act as an energy trap for reducing equivalents generated by critical reductases, thereby compromising the redox and energy metabolism in *G*. *lamblia* and other protozoa [10,11,72]. However, direct evidence for these mechanisms remains sparse, in part because it is difficult to separate causal from incidental events in nitro drug actions. Our data and another recent report [14] suggest that direct inactivation of critical proteins by adduction is another potential mechanism of action for nitro drugs in the anaerobic parasite *G*. *lamblia*. As an example, we found for ADI that the degree of drug adduction corresponded closely to the degree of enzyme inhibition at a particular drug concentration, suggesting a stoichiometric relationship between adduction and protein inactivation. Our studies subsequently revealed a mechanism by which nitro drug adduction of a specific cysteine residue of the protein can block the enzyme. These findings, combined with the demonstration that pharmacological inhibition of the enzyme was effective against trophozoites independent of Mz resistance, strongly suggests that ADI inactivation contributes to the giardicidal action of nitro drugs. Similarly, thioredoxin reductase is adducted and inactivated by a different nitro-heterocyclic compound [14], underlining that nitro drug adduction has direct inhibitory effects on target proteins. It must be noted, however, that our data do not prove that ADI inhibition is solely responsible for the drug actions, as the current study was not designed to address the relative importance of different potential action mechanism in the overall effects of nitro drugs.

While ADI must be considered a major candidate responsible for the nitro drug action in *G*. *lamblia*, our data also show that nitro drugs covalently bind to 160 other protein targets. Prior studies in *E*. *histolytica* and *G*. *lamblia* using two-dimensional gel electrophoresis and mass spectrometry also identified several nitro drug targets [10,11], although the numbers were lower compared to those identified by our click chemistry-based enrichment approach. This may be related to differential sensitivity of the different target identification strategies. Importantly, the current, click chemistry-based strategy for identification of adduction targets revealed many of the same targets, including thioredoxin reductase, alpha-11 giardin, elongation factor-1γ, pyruvate phosphate dikinase, and alcohol dehydrogenase, that were previously discovered [11], thus cross-validating the different strategies. Regardless of the exact number of targets, extensive functional studies will be needed to define the functional impact of adduction for each of these multiple targets in future studies. For now, one could speculate by simple extrapolation of the ADI data in *G*. *lamblia*, where one of three adduction events appeared to be functionally significant, and from Mz adduction studies in *T*. *vaginalis* [72], that target inactivation does not invariably occur after nitro drug adduction. However, many targets are nonetheless likely to be directly inactivated by nitro drugs, and could thereby contribute, perhaps synergistically, to the microbicidal activity of the drugs. Such multi-target activity, if it exists, might also explain why resistance to nitro drugs appears to occur largely on the level of prodrug activation rather than target avoidance [6,73], since we found that a similar spectrum of targets was adducted in resistant cells, albeit greater drug exposure was required to achieve similar levels of adduction. These results also suggest that nitro drugs able to exploit alternative prodrug activation pathways in *G*. *lamblia* will be active in MzR cells since their downstream targets are likely to remain available for inactivation. Prior studies with structurally diverse nitro drugs support this notion [29].

We found mass spectrometric evidence for two specific nitro drug adducts, 5-amino-4-thioether and 5-sulfinamide, on a limited number of cysteines in ADI, whereas the majority of cysteines in the enzyme were not adducted, indicating that nitro drug adduction exhibits structural and spatial specificity. Amino-thioether adducts on cysteines have also been reported in Mz- or tinidazole-treated *E*. *histolytica* thioredoxin and thioredoxin reductase [10], and have been identified as the major adducts in the in vitro reaction between cysteine and the 5-nitroimidazole, ronidazole, in the presence of either dithionite or rat liver microsomal enzymes as reducing agents [53]. Our studies did not reveal whether the two adduct structures have similar functional implications for the target proteins, but the physical dimensions of the adducts are likely to be similar so that any mechanism involving spatial obstruction, such as hindering substrate access to the catalytic site in ADI, is equally plausible for both adducts. We note that despite the substantial support for the C_8_H_9_N_3_ adduct being an amino-thioether, we cannot definitively exclude that this adduct is a sulfenamide. Although we are not aware of any published reports on sulfenamide adducts of 5-nitroimidazoles, sulfenamides have been isolated and identified as products in reactions between nitrosobenzenes and thiols [57,58] and upon reaction of glutathione with a hydroxylamino derivative from a 2-nitroimidazole [61].

Our target identification strategy uncovered several dozen potential drug targets in *G*. *lamblia*. This number occupies a middle ground of candidates suitable for further testing when compared to other drug development strategies. It is more focused than broad genomics screens (such as siRNA- or CRISPR/Cas9-based screens) which can reveal hundreds or even thousands of candidates, yet more extensive than hypothesis-driven pursuit of individual targets. Furthermore, by identifying candidate targets rather than chemical entities with antimicrobial activity, as done with broad, target-agnostic chemical library screens, our target identification strategy is more amenable to systematic, target-focused drug development. From these different perspectives, our strategy should be a useful addition to the armamentarium of antimicrobial drug development tools. Of course, the strategy can presently only be applied to microbes that are susceptible to 5-NI drugs, although this list contains a number of clinically important infectious agents beyond the model pathogen, *G*. *lamblia*, used for this work. For example, preliminary work with other pathogenic protozoa, *E*. *histolytica* and *T*. *vaginalis*, revealed similar numbers of adduction targets in their 5-NI drug adductomes. Together, our studies provide insights that can help focus new drug development efforts on a manageable number of promising targets, particularly for overcoming antimicrobial resistance in different pathogenic protozoa.

## Supporting information

S1 Table Biochemical detection of 5-NI drug adduction in *G*. *lamblia*. Trophozoites of Mz-sensitive (MzS, experiments 1–3) and congenic Mz-resistant (MzR) lines of *G*. *lamblia* WB (GL50803) were treated with Mz-alkyne for 2 h, after which cell lysates were prepared and reacted with azido-biotin using the click reaction. In a separate experiment, cell lysates were prepared from untreated *G*. *lamblia* WB trophozoites, and incubated with Mz-alkyne and dithionite as an external reducing system before reacting with azido-biotin by the click reaction. Biotin-labeled proteins were purified by streptavidin affinity chromatography, and identified by in situ trypsin digestion and subsequent LC-MS/MS analysis. Peptide spectral counts (SpC) were tabulated, and used to calculate the normalized spectral abundance factor (NSAF), which is shown as mean ± SD of three experiments with MzS cells, or as means for MzR cells and the cell lysate reaction. L designates the protein length in number of amino acids (aa). Gene ID refers to the *G*. *lamblia* WB genome (GL50803). Significances were calculated for each adducted gene product based on the NSAF values from the independent experiments in MzS cells relative to no adduction. All proteins with p<0.05 (entries 1–51) are also shown Table 1 in the main paper.
(PDF)
