# Supplementary material for: Click chemistry-facilitated comprehensive identification of proteins adducted by antimicrobial 5-nitroimidazoles for discovery of alternative drug targets against giardiasis
Source: PLoS Negl Trop Dis. 2020 Apr 17;14(4):e0008224. doi: 10.1371/journal.pntd.0008224 (PMC7190177; doi:10.1371/journal.pntd.0008224)
Supplement: S1 Table — Trophozoites of Mz-sensitive (MzS, experiments 1–3) and congenic Mz-resistant (MzR) lines of G. lamblia WB (GL50803) were treated with Mz-alkyne for 2 h, after which cell lysates were prepared and reacted with azido-biotin using the click reaction. In a separate experiment, cell lysates were prepared from untreated G. lamblia WB trophozoites, and incubated with Mz-alkyne and dithionite as an external reducing system before reacting with azido-biotin by the click reaction. Biotin-labeled proteins were purified by streptavidin affinity chromatography, and identified by in situ trypsin digestion and subsequent LC-MS/MS analysis. Peptide spectral counts (SpC) were tabulated, and used to calculate the normalized spectral abundance factor (NSAF), which is shown as mean ± SD of three experiments with MzS cells, or as means for MzR cells and the cell lysate reaction. L designates the protein length in number of amino acids (aa). Gene ID refers to the G. lamblia WB genome (GL50803). Significances were calculated for each adducted gene product based on the NSAF values from the independent experiments in MzS cells relative to no adduction. All proteins with p<0.05 (entries 1–51) are also shown Table 1 in the main paper. (PDF) [file pntd.0008224.s001.pdf]

Supplemental Table 1

Click chemistry-facilitated comprehensive identification of proteins adducted by antimicrobial 5-nitroimidazoles for discovery of alternative drug targets against giardiasis

Tineke Lauwaet, Yukiko Miyamoto, Sozaburo Ihara, Christine Le, Jarosław Kalisiak,

Keith Korthals, Majid Ghassemian, Diane K. Smith, K. Barry Sharpless,

Valery V. Fokin, and Lars Eckmann

Supplemental Table 1. Comprehensive analysis of proteins adducted by Mz-alkyne in *G. lamblia*

					MzS cells Exp 1		MzS cells Exp 2		MzS cells Exp 3		MzS cells			MzR cells		Cell lysate	
**No**	**Gene ID**	**Gene product**	**L (aa)**	**Function**	**SpC**	**NSAF x100**	**SpC**	**NSAF x100**	**SpC**	**NSAF x100**	**Mean**	**SD**	**p**	**SpC MzR**	**NSAF x100**	**SpC Invitro**	**NSAF x100**
1	10311	Ornithine carbamoyltransferase	327	Metabolism	17	5.65	22	5.84	11	5.10	5.63	0.41	0.001	10	10.82	9	7.63
2	14521	Peroxiredoxin 1	201	Redox	4	2.16	6	2.59	5	3.77	2.89	0.84	0.013	3	5.28	3	4.14
3	5810	Hypothetical protein	131	Hypothetical	3	2.49	3	1.99	3	3.47	2.69	0.76	0.013	0	0	1	2.12
4	16453	Carbamate kinase	316	Metabolism	7	2.41	9	2.47	6	2.88	2.63	0.25	0.002	4	4.48	5	4.39
5	112304	Elongation factor 1-alpha	442	Protein biosynthesis	8	1.97	12	2.36	9	3.09	2.51	0.57	0.008	6	4.80	6	3.77
6	16076	Peroxiredoxin 1	201	Redox	4	2.16	5	2.16	4	3.02	2.49	0.50	0.007	3	5.28	3	4.14
7	112103	Arginine deiminase	580	Metabolism	16	3.00	16	2.39	5	1.31	2.27	0.88	0.023	5	3.05	2	0.96
8	6687	Glyceraldehyde 3-phosphate dehydrogenase	336	Metabolism	8	2.59	9	2.32	3	1.35	2.13	0.67	0.016	2	2.11	5	4.13
9	103676	Alpha-tubulin	454	Cytoskeleton	8	1.92	7	1.34	8	2.67	2.01	0.67	0.018	5	3.90	5	3.05
10	10429	Wos2 protein	185	Chaperone	4	2.35	3	1.41	2	1.64	1.83	0.50	0.012	0	0	0	0
11	7110	Ubiquitin	82	Proteasome	1	1.33	2	2.12	1	1.85	1.79	0.41	0.009	0	0	0	0
12	14614	Eukaryotic translation initiation factor 5A	149	Protein biosynthesis	2	1.46	3	1.75	2	2.04	1.78	0.29	0.004	1	2.38	0	0
13	3910	Hypothetical protein	123	Hypothetical	2	1.77	3	2.12	1	1.23	1.74	0.46	0.011	0	0	0	0
14	6430	14-3-3 protein	248	Signaling	2	0.88	5	1.75	3	1.84	1.51	0.54	0.020	2	2.85	0	0
15	17153	Alpha-11 giardin	307	Cytoskeleton	3	1.06	4	1.13	4	1.98	1.41	0.51	0.021	2	2.31	2	1.81
16	21942	NADP-specific glutamate dehydrogenase	449	Metabolism	8	1.94	8	1.55	2	0.68	1.41	0.66	0.033	1	0.79	3	1.85
17	17163	Peptidyl-prolyl cis-trans isomerase B precursor	168	Protein folding	1	0.65	3	1.55	2	1.81	1.36	0.62	0.031	1	2.11	1	1.65
18	17547	Ribosomal protein L4	316	Protein biosynthesis	3	1.03	4	1.1	3	1.44	1.21	0.22	0.005	0	0	2	1.76
19	88765	Cytosolic HSP70	664	Chaperone	8	1.31	10	1.31	3	0.69	1.12	0.37	0.017	4	2.13	2	0.84
20	15869	GTP-binding nuclear protein RAN/TC4	226	Signaling	3	1.44	3	1.15	1	0.67	1.11	0.40	0.020	1	1.57	0	0
21	17327	Xaa-Pro dipeptidase	444	Proteasome	5	1.22	4	0.78	3	1.03	1.03	0.23	0.008	2	1.59	3	1.87
22	11118	Enolase	445	Metabolism	5	1.22	4	0.78	3	1.02	1.03	0.22	0.008	0	0		0
23	9909	Pyruvate, phosphate dikinase	884	Metabolism	12	1.48	9	0.88	4	0.69	1.03	0.42	0.025	2	0.80	6	1.88

24	21628	Hypothetical protein	383	Hypothetical	4	1.14	3	0.68	3	1.19	1.02	0.28	0.012	0	0	2	1.45
25	9779	UPL-1	310	Metabolism	3	1.05	3	0.84	2	0.98	0.97	0.11	0.002	0	0	1	0.89
26	15520	Ribosomal protein L21	159	Protein biosynthesis	2	1.37	1	0.55	1	0.95	0.97	0.42	0.028	1	2.23	2	3.49
27	17244	Ribosomal protein L7a	225	Protein biosynthesis	2	0.97	3	1.16	1	0.67	0.95	0.25	0.011	0	0	0	0
28	90872	Phosphoglycerate kinase	409	Metabolism	4	1.06	6	1.27	1	0.37	0.92	0.48	0.040	0	0	1	0.68
29	19436	Ribosomal protein L7	235	Protein biosynthesis	2	0.93	3	1.11	1	0.65	0.91	0.24	0.011	0	0	0	0
30	17054	Acidic ribosomal protein P0	326	Protein biosynthesis	2	0.67	2	0.53	3	1.40	0.88	0.47	0.042	1	1.09	0	0
31	10255	Translation initiation factor eIF-4A, putative	391	Protein biosynthesis	3	0.83	4	0.89	2	0.78	0.85	0.06	0.001	1	0.91	0	0
32	17060	Protein 21.1	623	Cytoskeleton	5	0.87	4	0.56	4	0.97	0.82	0.22	0.012	1	0.57	2	0.89
33	13864	Heat shock protein HSP 90-alpha	324	Chaperone	3	1.01	3	0.8	1	0.47	0.77	0.28	0.02	1	1.09	1	0.86
34	16431	Ribosomal protein L19	196	Protein biosynthesis	1	0.55	2	0.89	1	0.77	0.75	0.17	0.009	0	0	0	0
35	11654	Alpha-1 giardin	295	Cytoskeleton	3	1.11	2	0.59	1	0.51	0.75	0.33	0.03	1	1.20	3	2.82
36	17121	Bip	677	Chaperone	5	0.8	7	0.90	2	0.45	0.73	0.25	0.018	0	0	0	0
37	16525	Ribosomal protein L3	379	Protein biosynthesis	2	0.57	4	0.92	1	0.40	0.64	0.27	0.027	0	0	1	0.73
38	7766	Ribosomal protein SA	245	Protein biosynthesis	2	0.89	1	0.35	1	0.62	0.63	0.27	0.028	1	1.44	0	0
39	12102	Elongation factor 1-gamma	402	Protein biosynthesis	2	0.54	4	0.86	1	0.38	0.60	0.25	0.027	1	0.88	1	0.69
40	13747	C4 group specific protein	198		1	0.55	1	0.44	1	0.77	0.60	0.17	0.013	0	0	0	0
41	13561	Translation elongation factor	220	Protein biosynthesis	1	0.49	1	0.39	1	0.69	0.53	0.15	0.013	1	1.61	0	0
42	8118	Ribosomal protein S2	242	Protein biosynthesis	1	0.45	1	0.36	1	0.63	0.49	0.13	0.012	0	0	0	0
43	16086	Ribosomal protein L2	251	Protein biosynthesis	1	0.43	1	0.35	1	0.60	0.47	0.13	0.013	0	0	0	0
44	17400	Hypothetical protein	314	Hypothetical	1	0.35	2	0.55	1	0.48	0.47	0.11	0.009	0	0	0	0
45	11043	Fructose-bisphosphate aldolase	323	Metabolism	1	0.34	1	0.27	1	0.47	0.36	0.11	0.014	1	1.10	0	0
46	93358	Alcohol dehydrogenase	888	Metabolism	3	0.37	2	0.20	3	0.51	0.36	0.16	0.030	3	1.20	1	0.31
47	11390	Kinase, NEK	777	Signaling	1	0.14	4	0.45	2	0.39	0.33	0.17	0.038	1	0.46	0	0
48	15832	Aminoacyl-histidine dipeptidase	524	Proteasome	2	0.41	1	0.17	1	0.29	0.29	0.13	0.028	0	0	0	0
49	13500	TCP-1 chaperonin subunit theta	563	Chaperone	1	0.19	2	0.31	1	0.27	0.26	0.06	0.007	0	0	0	0

50	86511	Acyl-CoA synthetase	970	Metabolism	2	0.22	1	0.09	2	0.31	0.21	0.12	0.043	1	0.36	1	0.29
51	17063	Pyruvate-flavodoxin oxidoreductase	1199	Redox	1	0.09	1	0.07	1	0.13	0.10	0.03	0.016	0	0	2	0.46
52	101291	Beta tubulin	447	Cytoskeleton	4	0.97	5	0.97	9	3.05	1.69	1.21	0.069	7	5.54	5	3.10
53	16588	Hypothetical protein	122	Hypothetical	1	0.89	1	0.71	2	2.49	1.39	0.99	0.068	0	0	2	4.55
54	8064	Protein disulfide isomerase PDI5	134	Protein folding	3	2.43	0	0	1	1.13	1.21	1.24	0.117	1	2.64	2	4.14
55	8462	Ribosomal protein L27	135	Protein biosynthesis	0	0	3	1.93	1	1.12	1.04	0.99	0.106	0	0	1	2.05
56	14670	Protein disulfide isomerase PDI3	116	Protein folding	0	0	2	1.50	1	1.31	0.95	0.83	0.093	0	0	1	2.39
57	27925	Protein 21.1	786	Signaling	4	0.55	3	0.33	9	1.74	0.89	0.76	0.091	0	0	2	0.71
58	14620	Ribosomal protein S6	248	Protein biosynthesis	2	0.88	4	1.40	0	0	0.77	0.72	0.102	0	0	0	0
59	17090	Giardia trophozoite antigen GTA-1	181	Membrane	1	0.6	0	0	2	1.68	0.77	0.86	0.131	0	0	0	0
60	3593	Alcohol dehydrogenase lateral transfer candidate	407	Metabolism	5	1.34	3	0.64	0	0	0.67	0.68	0.115	0	0	0	0
61	9030	Protein 21.1	330	Signaling	1	0.33	1	0.26	3	1.38	0.67	0.63	0.104	0	0	0	0
62	8528	Hypothetical protein	528	Hypothetical	2	0.41	2	0.33	4	1.15	0.64	0.45	0.067	1	0.67	1	0.53
63	15409	Kinase, NEK	515	Signaling	1	0.21	1	0.17	5	1.47	0.62	0.75	0.144	2	1.37	4	2.15
64	23004	Hypothetical protein	163	Hypothetical	0	0	0	0	2	1.86	0.63	1.09	0.211	0	0	0	0
65	3643	70 kDa peptidylprolyl isomerase, putative	338	Protein biosynthesis	3	0.96	3	0.77	0	0	0.59	0.52	0.094	0	0	0	0
66	11301	Nucleoside diphosphate kinase	151	DNA dynamics	1	0.72	0	0	1	1.00	0.58	0.53	0.097	0	0	1	1.84
67	4652	Ribosomal protein S16	158	Protein biosynthesis	1	0.69	0	0	0	0	0.23	0.40	0.211	0	0	1	1.76
68	29487	Protein disulfide isomerase PDI1	234	Protein folding	1	0.46	3	1.11	0	0	0.54	0.57	0.123	0	0	0	0
69	14938	Ribosomal protein L12	182	Protein biosynthesis	1	0.60	2	0.95	0	0	0.53	0.49	0.102	0	0	0	0
70	5845	Ribosomal protein S8	174	Protein biosynthesis	1	0.62	0	0	1	0.87	0.51	0.45	0.097	0	0	1	1.59
71	6289	FixW protein, putative	133	Redox	1	0.82	1	0.65	0	0	0.5	0.44	0.094	0	0	0	0
72	9355	Hypothetical protein	134	Hypothetical	1	0.81	1	0.65	0	0	0.50	0.44	0.094	0	0	0	0
73	114787	Alpha-7.3 giardin	295	Cytoskeleton	0	0	5	1.47	0	0	0.50	0.87	0.211	2	2.40	0	0
74	15411	Kinase, NEK-frag	706	Signaling	1	0.15	0	0	6	1.29	0.49	0.71	0.178	0	0	0	0
75	1695	Rab11	216	Signaling	2	1.01	1	0.40	0	0	0.48	0.51	0.124	0	0	0	0

76	7870	Ribosomal protein L23A	141	Protein biosynthesis	1	0.77	1	0.62	0	0	0.47	0.41	0.094	0	0	0	0
77	4547	Ribosomal protein S9	189	Protein biosynthesis	1	0.58	0	0	1	0.80	0.47	0.42	0.097	0	0	2	2.94
78	11354	Hypothetical protein	281	Hypothetical	2	0.77	2	0.62	0	0	0.47	0.42	0.094	0	0	0	0
79	5593	Ribosomal protein L11	173	Protein biosynthesis	0	0	1	0.50	1	0.88	0.47	0.45	0.106	0	0	0	0
80	1345	Ribosomal protein L10a	221	Protein biosynthesis	2	0.98	1	0.39	0	0	0.47	0.50	0.125	0	0	0	0
81	103713	Protein disulfide isomerase PDI4	354	Protein folding	2	0.61	3	0.74	0	0	0.46	0.40	0.093	0	0	0	0
82	14993	Pyrophosphate-fructose 6-phosphate 1-phosphotransferase alpha subunit	544	Metabolism	4	0.80	3	0.48	0	0	0.43	0.41	0.104	0	0	1	0.51
83	9413	Protein disulfide isomerase PDI2	449	Protein folding	3	0.73	1	0.19	1	0.34	0.43	0.28	0.059	1	0.79	1	0.62
84	12981	Ribosomal protein S5	190	Protein biosynthesis	0	0	1	0.46	1	0.80	0.43	0.41	0.105	0	0	0	0
85	98054	Heat shock protein HSP 90-alpha	376	Chaperone	0	0	2	0.46	2	0.81	0.43	0.41	0.106	0	0	0	0
86	13608	Acetyl-CoA synthetase	726	Metabolism	5	0.75	4	0.48	0	0	0.42	0.39	0.101	0	0	1	0.38
87	137716	Axoneme-associated protein GASP-180	1585	Cytoskeleton	0	0	0	0	13	1.24	0.42	0.73	0.211	5	1.12	5	0.88
88	15383	Peroxiredoxin 1	246	Redox	1	0.44	2	0.71	0	0	0.39	0.36	0.102	0	0	0	0
89	14285	Malic enzyme	557	Metabolism	3	0.59	3	0.47	0	0	0.36	0.32	0.094	0	0	0	0
90	16265	Ribosomal protein S3a	248	Protein biosynthesis	0	0	3	1.05	0	0	0.36	0.62	0.211	0	0	0	0
91	15410	Ser/Thr protein kinase	285	Signaling	0	0	0	0	2	1.06	0.36	0.62	0.211	0	0	0	0
92	14586	VSP with INR	733	Membrane	0	0	0	0	5	1.03	0.35	0.61	0.211	0	0	1	0.38
93	17570	Elongation factor 2	898	Protein biosynthesis	5	0.61	4	0.39	0	0	0.34	0.31	0.102	1	0.39	0	0
94	9827	Thioredoxin reductase	314	Redox	2	0.69	1	0.28	0	0	0.33	0.35	0.125	0	0	1	0.88
95	17230	Gamma giardin	311	Cytoskeleton	0	0	0	0	2	0.98	0.33	0.57	0.211	2	2.28	1	0.89
96	15214	Ser/Thr phosphatase	308	Signaling	1	0.35	2	0.56	0	0	0.31	0.29	0.102	0	0	0	0
97	3331	Malate dehydrogenase	331	Metabolism	2	0.66	1	0.26	0	0	0.31	0.34	0.124	0	0	0	0
98	27310	Stress-induced-phosphoprotein 1	587	Chaperone	1	0.19	5	0.74	0	0	0.31	0.39	0.149	1	0.60	0	0
99	15567	Rab2a	214	Signaling	1	0.51	1	0.41	0	0	0.31	0.27	0.095	0	0	0	0
100	17055	Spindle pole protein, putative	554	Cytoskeleton	2	0.39	3	0.47	0	0	0.29	0.26	0.093	0	0	0	0
101	7195	Glutamate synthase	910	Metabolism	4	0.48	4	0.38	0	0	0.29	0.26	0.094	0	0	0	0

102	16412	Heat-shock protein, putative	845	Chaperone	1	0.13	7	0.72	0	0	0.29	0.39	0.165	0	0	0	0
103	9808	Chaperone protein DnaJ	409	Chaperone	0	0	4	0.85	0	0	0.29	0.50	0.211	0	0	0	0
104	114609	Pyruvate-flavodoxin oxidoreductase	1253	Redox	6	0.52	4	0.28	0	0	0.27	0.27	0.110	0	0	0	0
105	5883	Hypothetical protein	562	Hypothetical	0	0	0	0	3	0.81	0.27	0.47	0.211	0	0	0	0
106	9861	Hypothetical protein	385	Hypothetical	2	0.56	1	0.23	0	0	0.27	0.29	0.124	1	0.92	0	0
107	7244	Hypothetical protein	192	Hypothetical	0	0	0	0	1	0.79	0.27	0.46	0.211	1	1.84	1	1.44
108	12150	Alanine aminotransferase, putative	521	Protein biosynthesis	2	0.42	2	0.33	0	0	0.25	0.22	0.094	1	0.68	0	0
109	10358	A-type flavoprotein lateral transfer candidate	414	Redox	2	0.53	1	0.21	0	0	0.25	0.27	0.125	0	0	0	0
110	14373	Dynamin	732	Protein transport	0	0	1	0.12	3	0.62	0.25	0.33	0.162	2	0.97	3	1.14
111	24321	Kinase, NEK	288	Signaling	2	0.75	0	0	0	0	0.26	0.44	0.211	0	0	0	0
112	9704	Transketolase	719	Metabolism	2	0.30	3	0.36	0	0	0.23	0.20	0.093	0	0	0	0
113	103373	Alpha-7.1 giardin	388	Cytoskeleton	1	0.28	0	0	1	0.39	0.23	0.21	0.097	2	1.82	0	0
114	114119	Alpha-7.2 giardin	388	Cytoskeleton	1	0.28	0	0	1	0.39	0.23	0.21	0.097	2	1.82	0	0
115	16867	AAA family ATPase	870	ATPase	3	0.37	3	0.3	0	0	0.23	0.20	0.094	1	0.41	0	0
116	13272	Hypothetical protein	367	Hypothetical	0	0	1	0.24	1	0.41	0.22	0.21	0.106	0	0	0	0
117	9719	NADH oxidase	429	Metabolism	1	0.25	2	0.40	0	0	0.22	0.21	0.102	0	0	0	0
118	10521	Arginyl-tRNA synthetase	621	Protein biosynthesis	1	0.18	0	0	2	0.49	0.22	0.25	0.129	0	0	2	0.89
119	7260	Aldose reductase	313	Metabolism	1	0.35	1	0.28	0	0	0.21	0.19	0.094	0	0	0	0
120	15427	Giardia trophozoite antigen GTA-2	236	Membrane	0	0	0	0	1	0.64	0.22	0.38	0.211	1	1.50	0	0
121	15297	Ribokinase	344	Metabolism	2	0.63	0	0	0	0	0.21	0.37	0.211	0	0	0	0
122	7588	Serine/threonine protein phosphatase 7	418	Signaling	0	0	3	0.62	0	0	0.21	0.37	0.211	0	0	0	0
123	12216	Vacuolar ATP synthase subunit B	497	Metabolism	2	0.44	1	0.17	0	0	0.21	0.23	0.125	0	0	0	0
124	5942	Polyadenylate-binding protein, putative	442	Protein biosynthesis	0	0	3	0.59	0	0	0.20	0.35	0.211	0	0	0	0
125	15097	Alpha-14 giardin	337	Cytoskeleton	1	0.32	1	0.26	0	0	0.20	0.17	0.095	0	0	0	0
126	15551	Ribosomal protein S18	154	Protein biosynthesis	0	0	1	0.56	0	0	0.19	0.33	0.211	0	0	1	1.80
127	16779	Cathepsin B precursor	298	Proteasome	0	0	2	0.58	0	0	0.20	0.34	0.211	0	0	0	0
128	16745	Axoneme-associated	1039	Cytoskeleton	0	0	0	0	4	0.58	0.20	0.34	0.211	0	0	0	0

		protein GASP-180														
129	17551	Protein 21.1	1074	Cytoskeleton	0	0	0	0	4	0.57	0.19	0.33	0.211	0	0	1	0.26
130	16353	Hypothetical protein	755	Hypothetical	2	0.29	2	0.23	0	0	0.17	0.15	0.094	0	0	0	0
131	17143	Pyruvate kinase	553	Metabolism	1	0.20	2	0.31	0	0	0.17	0.16	0.102	0	0	0	0
132	10661	Ubiquitin-conjugating enzyme E1	1092	Proteasome	2	0.20	4	0.32	0	0	0.17	0.16	0.102	0	0	0	0
133	86600	Methionine aminopeptidase	420	Proteasome	1	0.26	1	0.21	0	0	0.16	0.14	0.094	0	0	0	0
134	112681	NSF	830	Endocytic pathway	2	0.26	2	0.21	0	0	0.16	0.14	0.095	0	0	0	0
135	8822	2,3-bisphosphoglycerate-independent phosphoglycerate mutase	589	Metabolism	1	0.18	2	0.29	0	0	0.16	0.15	0.102	0	0	0	0
136	11950	Ribosomal protein L18	179	Protein biosynthesis	0	0	1	0.48	0	0	0.17	0.29	0.211	0	0	1	1.55
137	14859	Protein 21.1	925	Cytoskeleton	0	0	0	0	3	0.49	0.17	0.29	0.211	0	0	1	0.30
138	15148	Chaperone protein dnaJ	614	Chaperone	1	0.18	2	0.28	0	0	0.16	0.15	0.102	0	0	0	0
139	7532	Vacuolar ATP synthase catalytic subunit A	655	Metabolism	2	0.33	1	0.13	0	0	0.16	0.17	0.123	0	0	0	0
140	15048	ATP-dependent RNA helicase-like protein	656	RNA biosynthesis	2	0.33	1	0.13	0	0	0.16	0.17	0.123	0	0	0	0
141	7789	ADP-ribosylation factor	191	Signaling	0	0	1	0.45	0	0	0.15	0.27	0.211	1	1.85	0	0
142	16667	Acyl-CoA synthetase	905	Metabolism	1	0.12	3	0.29	0	0	0.14	0.15	0.123	0	0	0	0
143	5795	Leucine-rich repeat protein 1 virus receptor protein	749	Hypothetical	2	0.29	1	0.12	0	0	0.14	0.15	0.125	0	0	0	0
144	101501	Seryl-tRNA synthetase	457	Protein biosynthesis	0	0	2	0.38	0	0	0.13	0.23	0.211	0	0	0	0
145	113553	Kinase, NEK	650	Signaling	0	0	1	0.13	1	0.23	0.13	0.12	0.105	0	0	0	0
146	21423	Beta adaptin	1132	Protein transport	0	0	3	0.23	1	0.13	0.12	0.12	0.103	0	0	0	0
147	11380	Hypothetical protein	493	Hypothetical	0	0	2	0.35	0	0	0.12	0.21	0.211	0	0	0	0
148	16343	Median body protein	857	Cytoskeleton	0	0	0	0	2	0.35	0.12	0.21	0.211	0	0	0	0
149	9115	Glucose-6-phosphate isomerase	584	Metabolism	1	0.19	1	0.15	0	0	0.11	0.1	0.095	0	0	0	0
150	16824	Kinase, NEK	823	Signaling	1	0.13	2	0.21	0	0	0.12	0.11	0.105	0	0	0	0
151	101278	Hypothetical protein	1330	Hypothetical	2	0.16	2	0.13	0	0	0.10	0.09	0.095	0	0	1	0.21

152	8805	Kinase, SCY1	936	Signaling	1	0.12	2	0.19	0	0	0.10	0.10	0.102	0	0	0	0
153	16322	Neurogenic locus Notch protein precursor	592	Signaling	0	0	2	0.29	0	0	0.10	0.17	0.211	0	0	0	0
154	21118	Long chain fatty acid CoA ligase 5	765	Metabolism	1	0.14	1	0.11	0	0	0.09	0.08	0.093	0	0	0	0
155	17304	Alpha adaptin	783	Endocytic pathway	1	0.14	1	0.11	0	0	0.08	0.07	0.095	0	0	0	0
156	30476	Long chain fatty acid CoA ligase 4	804	Metabolism	0	0	2	0.22	0	0	0.07	0.13	0.211	0	0	0	0
157	9062	Long chain fatty acid CoA ligase 5	853	Metabolism	0	0	2	0.20	0	0	0.07	0.12	0.211	0	0	0	0
158	102101	Kinesin-3	1026	Cytoskeleton	1	0.11	1	0.08	0	0	0.07	0.06	0.094	0	0	0	0
159	96460	Alanyl-tRNA synthetase	969	Protein biosynthesis	0	0	2	0.18	0	0	0.06	0.10	0.211	0	0	0	0
160	15317	High cysteine membrane protein Group 1	1615	Membrane	0	0	2	0.11	0	0	0.04	0.06	0.211	0	0	0	0
161	9183	Hypothetical protein	1904	Hypothetical	1	0.06	1	0.05	0	0	0.04	0.03	0.093	0	0	4	0.58
				Total SpC:	330		406		233					104		125	

Trophozoites of Mz-sensitive (MzS, experiments 1-3) and congenic Mz-resistant (MzR) lines of *G. lamblia* WB (GL50803) were treated with Mz-alkyne for 2 h, after which cell lysates were prepared and reacted with azido-biotin using the click reaction. In a separate experiment, cell lysates were prepared from untreated *G. lamblia* WB trophozoites, and incubated with Mz-alkyne and dithionite as an external reducing system before reacting with azido-biotin by the click reaction. Biotin-labeled proteins were purified by streptavidin affinity chromatography, and identified by in situ trypsin digestion and subsequent LC-MS/MS analysis. Peptide spectral counts (SpC) were tabulated, and used to calculate the normalized spectral abundance factor (NSAF), which is shown as mean ± SD of three experiments with MzS cells, or as means for MzR cells and the cell lysate reaction. L designates the protein length in number of amino acids (aa). Gene ID refers to the *G. lamblia* WB genome (GL50803). Significances were calculated for each adducted gene product based on the NSAF values from the independent experiments in MzS cells relative to no adduction. All proteins with $p<0.05$ (entries 1-51) are also shown Table 1 in the main paper.
